# PARP Inhibitor Sensitivity in Tumors Harboring Non-BRCA Homologous Recombination Gene Alterations: Current Evidence Across Ovarian, Breast, Prostate, and Pancreatic Cancers

**DOI:** 10.3390/ijms27156754

**Published:** 2026-07-28

**Authors:** Elizabeth Santana dos Santos, André Luiz Cicilini, Maria Fernanda Evangelista Simões, Maria Baz, Sandrine M. Caputo, Etienne Rouleau

**Affiliations:** 1 Department of Clinical Oncology, A.C. Camargo Cancer Center, São Paulo 01509-010, Brazil; elizabeth.santanadossantos@gmail.com (E.S.d.S.); andre.cicilini@accamargo.org.br (A.L.C.); 2 Department of Oncogenetics, Hospital Sírio Libanês, São Paulo 01308-050, Brazil; 3 Department of Clinical Oncology, Hospital Sírio Libanês, São Paulo 01308-050, Brazil; mf_simoes@hotmail.com; 4 Gustave Roussy Cancer Genetics Laboratory, Department of Medical Biology and Pathology, 94800 Villejuif, France; maria.baz.baz@hotmail.com; 5 Department of Genetics, Institut Curie, Paris Sciences and Lettres (PSL) University, 75005 Paris, France; sandrine.caputo@curie.fr

**Keywords:** neoplasms, prostate cancer, breast cancer, ovarian cancer, pancreatic cancer, PARP inhibitors, homologous recombination deficiency, non-*BRCA HR* genes, HRR genes, DNA repair

## Abstract

Poly(ADP-ribose) polymerase inhibitors (PARPis) have demonstrated remarkable efficacy in tumors carrying *BRCA1/2* pathogenic variants (PVs) through the mechanism of synthetic lethality. PVs in other homologous recombination (HR) genes may also impair homologous recombination repair and confer sensitivity to PARPis; however, their predictive value remains uncertain and appears to vary according to the affected gene and tumor type. We review and critically discuss the current evidence regarding the predictive value of non-*BRCA HR* gene pathogenic variants as biomarkers of PARPi sensitivity across ovarian, breast, prostate, and pancreatic cancers. A narrative review was conducted between October 2023 and December 2025, first identifying pivotal clinical trials of PARP inhibitors across ovarian, breast, prostate, and pancreatic cancers, followed by a targeted search of PubMed, Embase, Web of Science, and Google Scholar for relevant preclinical and clinical studies. Seventeen clinical studies and multiple preclinical reports were analyzed regarding genomic frequency, HRD association, and treatment response. Preclinical studies consistently demonstrated increased PARPi sensitivity in models with alterations in several non-*BRCA HR* genes. Clinical evidence, however, was heterogeneous. *PALB2* demonstrated the strongest and most consistent association with PARPi benefit across tumor types, while *RAD51C* and *RAD51D* also showed clinically meaningful activity, particularly in ovarian cancer. In contrast, evidence supporting PARPi sensitivity in tumors harboring *ATM*, *CHEK2*, *CDK12*, and several other HR gene alterations remained limited or inconsistent. Differences in gene function, biallelic inactivation, variant type, and current limitations of HRD companion diagnostic assays likely contribute to the observed variability in clinical response. Non-*BRCA HR* gene alterations represent promising predictive biomarkers for PARPi therapy but should not be considered a homogeneous group. Future biomarker-driven studies integrating comprehensive genomic profiling and functional assessment of homologous recombination deficiency are needed to refine patient selection and optimize the clinical application of PARPis beyond *BRCA1/2*-associated cancers.

## 1. Introduction

The homologous recombination (HR) pathway is a high-fidelity DNA repair mechanism that enables accurate repair of DNA double-strand breaks and maintenance of genomic stability. Among the key genes involved in this pathway, *BRCA1* and *BRCA2* genes are the most extensively characterized and are frequently altered in hereditary and sporadic cancers [1]. Germline pathogenic variants (PVs) affecting one of these two genes increase the risk of developing cancer, particularly ovarian, breast, prostate and pancreatic cancers [2]. The proportion of HR-deficient homologous recombination deficiency (HRD) ovarian cancers is close to 50%: 20% with *BRCA1/2* pathogenic variants, 10% with *BRCA1* promoter methylation and 20% for other reasons [3]. The proportion of breast, prostate and pancreatic HRD cancers is about 21%, 10% and about 15%, respectively. To perform DNA repair, *BRCA1* and *BRCA2* and other HR genes, such as *PALB2*, *RAD51C/D*, *ATM*, *CHEK2*, are involved in a common pathway [4,5]. PVs in these genes may also lead to an HRD phenotype. PVs in *other HR* genes are also associated with cancer, but to a lesser extent [1,6,7,8,9,10,11]. The frequency of somatic *HR gene* PVs other than *BRCA1/2* in ovarian, breast, prostate and pancreatic cancers ranges from 5 to 10% [12,13]. In addition, germline PVs affecting HR genes have been identified in ~30% of patients with familial breast and ovarian cancer [14] and in 11% and 16% of metastatic prostate and pancreatic cancer patients, respectively [15,16]. Promoter methylation could also explain the HRD phenotype. Methylation of the *BRCA1* promoter remains significant only in breast and ovarian cancer (10–15%), and a few rare cases of methylation of the *RAD51C* promoter have also been reported in breast and ovarian cancers [17,18,19,20,21,22]. However, methylation is a rare event in the other cancers [10,17,19]. On the remaining proportion, PVs in other HR genes could explain the HRD phenotype.

**Figure 1:**
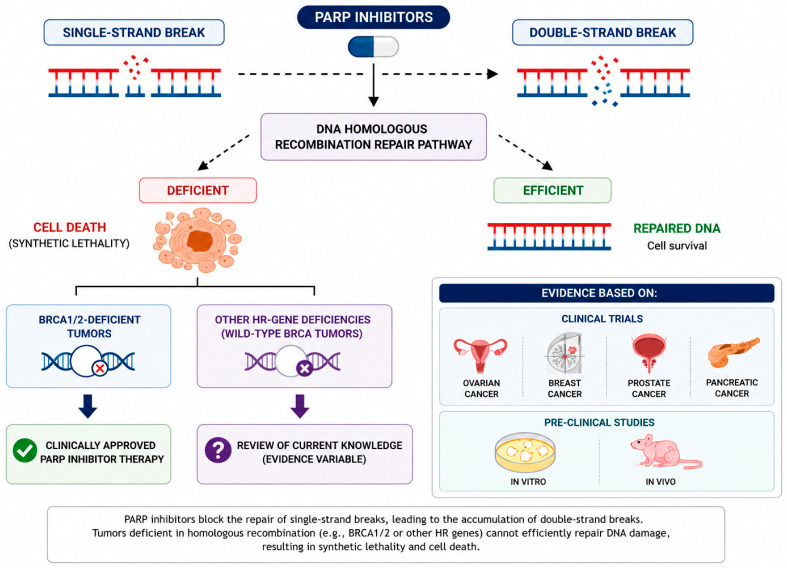
**Synthetic lethality induced by PARP inhibition in homologous recombination deficient tumors.** PARP inhibition leads to the accumulation of DNA damage that cannot be efficiently repaired in homologous recombination deficient cells, resulting in synthetic lethality, while homologous recombination proficient cells survive through effective DNA repair.

It is widely known that HRD tumors exhibit increased sensitivity to platinum agents because of the accumulation of unrepaired DNA damage leading to apoptosis [23]. PARPs are a family of enzymes involved in different cellular pathways, including DNA single-strand break repair. They trigger a cascade of events in response to single-stranded DNA breaks, leading to the recruitment of repair factors. It has been shown that HRD tumors rely on PARPs for survival [24]. Therefore, concurrent inhibition of PARP enzymes along with HRD results in an accumulation of DNA damage and genomic instability, which is lethal to cancer cells (Figure 1). This has been confirmed in preclinical studies as well as in clinical trials and has led to the development of PARPis as promising targeted therapy [25] and FDA approval of Olaparib, Niraparib, Talozaparib and Rucaparib for treatment of breast, ovarian, prostate and pancreatic cancers. However, most clinical trials to date have primarily focused on *BRCA1/2* PVs, and functional and clinical evidence regarding PARPi efficacy in tumors with PVs in non-*BRCA HR* genes remains limited (Figure 1).

Biomarker testing is critical to identify patients most likely to benefit from PARPis and to guide treatment decisions. For ovarian cancers in addition to *BRCA1/2* screening, determining HRD status (signature) is equally important to guide treatment decisions as PARPi maintenance therapy showed the greatest benefit for patients with a *BRCA1/2* PV or for those who tested positive for HRD. Two FDA-approved commercial tests are available for determining ovarian cancer HRD status: Myriad MyChoice^®^ CDx tests for the presence of a *BRCA1/2* PV and/or genomic instability (LOH, telomeric allelic imbalance, and large-scale state transitions), and FoundationOne CDxTM tests for the presence of a *BRCA1/2* PV and LOH. Academic-laboratory-developed tests are also an alternative to commercial assays [26,27,28,29,30,31,32]. However, for breast, prostate and pancreatic cancers HRD phenotype analysis is not part of the daily routine, and the choice of using PARPis is still limited to cases with identified *BRCA1/2* PVs [33]. The predictive potential of non-*BRCA HR* PVs (e.g., PVs in *RAD51C*, *RAD51D*, *BRIP1*, *PALB2*, *NBN*, *ATM*, *CHEK2*, *CDK12*) has been evaluated as exploratory analysis of clinical trial data, but no definitive conclusion can be reached so far.

The review aims to present the current evidence regarding the potential role of non-*BRCA HR* gene PVs as predictive biomarkers of response to PARPi therapy for ovarian, breast, prostate, and pancreatic cancers.

## 2. Methodology

### 2.1. Literature Search Strategy

This study was conducted as a narrative review aimed at summarizing and critically discussing the available evidence regarding the predictive value of pathogenic variants in non-*BRCA* homologous recombination (HR) genes for response to poly(ADP-ribose) polymerase inhibitors (PARPis) across ovarian, breast, prostate, and pancreatic cancers.

**Figure 2:**
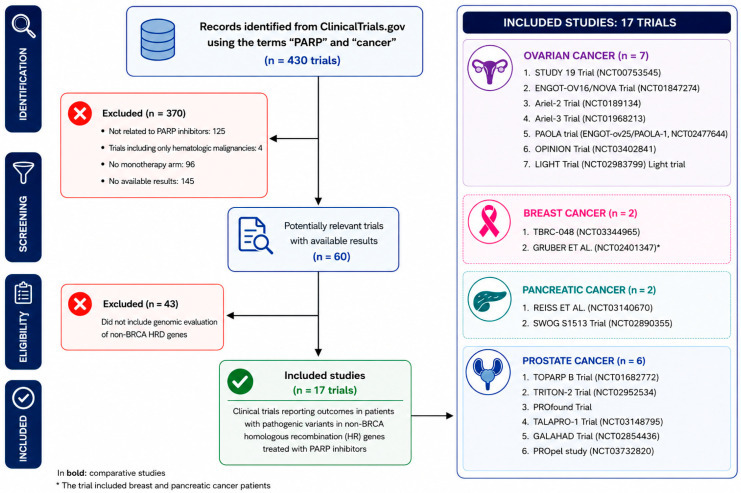
**Summary of the literature review used in this report.** Selection of clinical phase I, II or III studies investigating PARPi monotherapy for treatment or ovarian, breast, prostate and pancreatic cancer patients harboring non-*BRCA HR* gene pathogenic variants.

A structured literature search was performed between October 2023 and December 2025 using PubMed, Embase, Web of Science, and Google Scholar. The search combined Medical Subject Headings (MeSH) and free-text terms related to PARP inhibitors, homologous recombination deficiency (HRD), DNA damage response (DDR), and homologous recombination genes. Search terms included combinations of “PARP inhibitors”, “homologous recombination deficiency”, “DNA damage repair”, “non-BRCA”, “PALB2”, “RAD51C”, “RAD51D”, “BRIP1”, “ATM”, “CHEK2”, “CDK12”, “NBN”, “ovarian cancer”, “breast cancer”, “prostate cancer”, and “pancreatic cancer”. Reference lists of relevant publications and recent review articles were also manually screened to identify additional eligible studies (Figure 2).

### 2.2. Study Selection

Publications were selected according to their relevance to the objectives of this review. Eligible studies included preclinical investigations, prospective and retrospective clinical studies, translational research, biomarker analyses, and clinical trials evaluating the efficacy of PARPis in tumors harboring pathogenic variants in non-*BRCA HR* genes. High-quality review articles, consensus statements, and current clinical guidelines were also consulted to provide background information and contextualize the available evidence.

**Figure 3:**
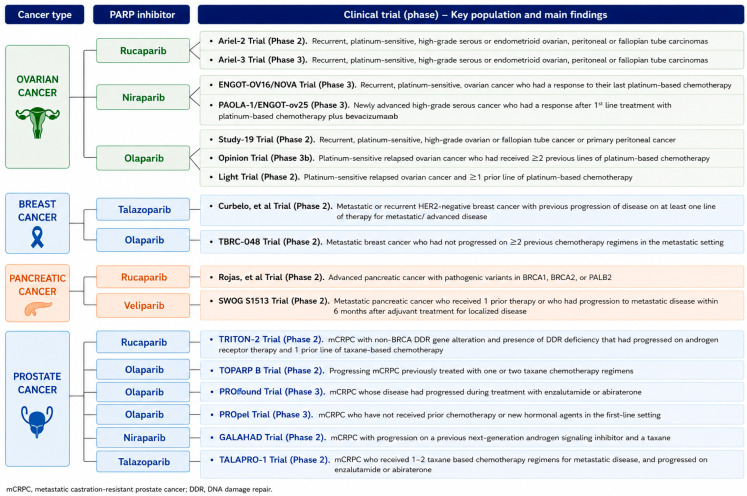
**Description of clinical trials according to the primary tumor.** Trials are organized according to primary tumor type, PARP inhibitor, trial phase, target population, and main clinical characteristics.

When multiple publications reported results from the same clinical trial, the most recent or comprehensive report was preferentially included. Greater emphasis was placed on studies reporting clinically meaningful outcomes, including objective response rate (ORR), progression-free survival (PFS), overall survival (OS), duration of response (DoR), and clinical benefit rate (CBR), whenever available. Considering the inclusion criteria, 17 clinical studies were selected for inclusion (Figure 2 and Figure 3). We will initially present the frequency of genomic alterations and preclinical data in the Section 3, followed by the presentation of clinical data available so far.

### 2.3. Data Synthesis

The evidence was synthesized narratively and organized according to individual HR genes and tumor type. Preclinical and clinical evidence were evaluated separately to distinguish experimental findings from clinically validated outcomes. Particular attention was given to the consistency of evidence supporting each non-*BRCA HR* gene as a predictive biomarker of PARPi sensitivity, as well as to factors potentially contributing to heterogeneous treatment responses across studies, including differences in study design, patient populations, molecular alterations, and HRD assessment methods.

As this work was designed as a narrative review, no formal systematic review protocol, prospective registration, or PRISMA-guided study selection process was performed [34].

## 3. Gene Review and Preclinical Studies

**Figure 4:**
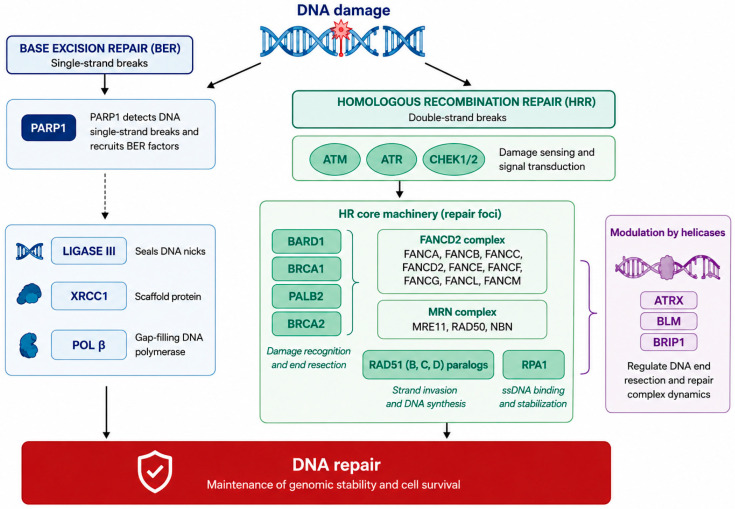
**Overview of DNA damage repair pathways relevant to PARPi sensitivity and HRD.** DNA damage is repaired through two major pathways: base excision repair (BER), responsible for repairing single-strand breaks in a PARP1-dependent manner, and homologous recombination repair (HRR), which repairs double-strand breaks through the coordinated action of DNA damage sensors (*ATM*, *ATR*, *CHEK1/2*), core HR proteins (*BRCA1*, *BRCA2*, *PALB2*, *BARD1*, *RAD51* paralogs, FANC complex, MRN complex, and *RPA1*), and regulatory helicases (*ATRX*, *BLM*, and *BRIP1*). Defects in HRR components result in homologous recombination deficiency (HRD), impairing DNA repair and increasing tumor susceptibility to PARP inhibitors through synthetic lethality. The figure highlights the principal molecular components involved in both repair pathways and their contribution to the maintenance of genomic stability.

**Figure 5:**
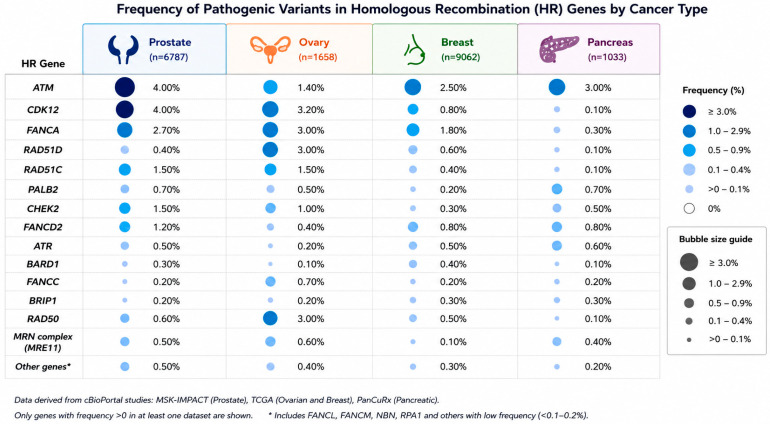
Frequency of homologous recombination repair (HRR) gene alterations across prostate, ovarian, breast, and pancreatic cancers. Higher frequencies were observed in key genes such as *BRCA1*, *BRCA2*, and *ATM*, with variability across tumor types, reflecting differences in molecular profiles and HRR pathway dependency.

This section summarizes the principal genes (Figure 4) identified across the included studies, most of which are related to HR, and lists them alphabetically. Table 1 and Figure 5 present the frequency of somatic alterations in *HR* genes. Preclinical evidence and the functional impact of PVs on the HR pathway are presented in Table 2.

***ATM***

Ataxia telangiectasia mutated (*ATM*) is a protein kinase with a key role in cell signaling in response to DNA double-stranded breaks and in recruiting proteins involved in repair [63,64,65,66]. *ATM* gene is associated with autosomal dominant predisposition to breast, pancreatic and prostate cancers [67,68,69,70]. For ovarian cancer, lifetime absolute risk is less prominent, ranging from 2 to 3%. Preclinical models have shown that ATM depletion sensitizes breast cancer cells to PARP inhibition, suggesting a potential role of PARPis for treatment of breast cancers low in ATM protein expression, such as those arising in mutant *ATM* heterozygous carriers [40,71]. In agreement with this, synthetic lethality between PARPis and *ATM* PVs has been reported in cells of patients with ataxia telangiectasia [72]. Furthermore, it was shown that olaparib induces significant killing of ATM-deficient cells with a significant correlation between reduction in ATM expression and sensitivity to the PARPis [73,74]. Despite this, *ATM* pathogenic variants are not consistently associated with an HRD genomic signature in ovarian cancer and, similarly, this non-association has also been observed in breast and prostate cancers, where clinical benefit from PARP inhibition remains heterogeneous [35,75,76].

***ATR***

ATR is a serine/threonine kinase and a DNA damage sensor. It plays a role in the HR pathway by phosphorylating and activating several proteins (*BRCA1*, *CHEK1*, *MCM2*, *RAD17*, *RPA2*, *SMC1* and *p53/TP53*) responsible for the inhibition of mitosis and replication [77,78]. No association between hereditary cancers and *ATR* PVs has been validated to date. Somatic PVs have been reported in 0.5% of breast and prostate cancers and in 0.2% and 0.6% in ovarian and pancreatic cancers, respectively [79,80]. Preclinical studies have shown that loss of function of this gene is associated with higher sensitivity to PARPis [36,38,44,45,46]. Despite this, the HRD status is not associated with *ATR* PVs in ovarian cancer [35].

***ATRX***

The ATRX protein is a chromatin remodeling factor, initially described as a putative helicase protein due to sequence homology with the DNA repair and recombination RAD54 protein. ATRX is a member of the SNF2 subgroup of the SWI/SNF protein superfamily. It plays a role in suppressing proteins of the ATM pathway by decreasing histone H3K9me3 [77,81]. No association between any hereditary cancer and this gene has been validated to date. However, somatic PVs have been reported in 0.4% of breast and prostate cancers, in 0.7% and 0.1% of ovarian and pancreatic cancers, respectively [79,80]. CRISPR-based studies revealed that loss of ATRX confers sensitivity to PARP inhibitors [82]. *ATRX* PVs were not associated with HRD status in ovarian cancer [35].

***BARD1***

*BARD1* is an E3 ubiquitin-protein ligase which interacts with *BRCA1* via its N-terminal RING finger domains to form a heterodimer essential for *BRCA1* stability [77,83]. Germline PVs in *BARD1* confer a twofold higher risk of breast cancer development [84,85,86]. Somatic PVs have been reported in 0.4% and 0.3% of breast and prostate cancers, respectively, and in 0.1% of ovarian and pancreatic cancers [79,80]. Preclinical studies have shown that loss of function of BARD1 confers higher sensitivity of tumor cells to PARPis [47]. The HRD status is not associated with *BARD1* PVs in ovarian cancer sample alone [35].

***BLM***

*BLM* is an ATP-dependent DNA helicase that unwinds and resects the 5′ end of DNA during double-stranded break repair [77,87]. No definitive association between any hereditary cancer and this gene has been validated to date [84]. Somatic PVs have been described in 0.3% and 0.2% of breast and prostate cancers, respectively, and in 0.4% of ovarian and pancreatic cancers [79,80]. PVs in this gene conferred higher sensitivity of tumor cells to PARPis in two preclinical studies [39,48]. The HRD status is associated with *BLM* PVs in ovarian cancer [35].

***BRIP1***

*BRIP1* (BACH1) is a 5′ to 3′ DNA helicase and DNA-dependent ATPase. It has an important function in the HR pathway by binding BRCA1 by its BRCT repeat domain [77,88,89]. Germline PVs affecting *BRIP1* may increase the risk of breast cancer development 1.5-fold and ovarian cancer 5-fold compared to the normal population [84]. Somatic PVs have been reported in 0.3% of breast and pancreatic cancers, and in 0.2% of ovarian and prostate cancers [79,80]. Preclinical studies have shown that loss of function of BRIP1 confers higher sensitivity of cancer cells to PARPis [49,90]. *BRIP1* PVs have been associated with HRD status in ovarian cancer [35].

***CDK12***

*CDK12* is a cyclin-dependent kinase and a transcription factor involved in the HR pathway. It regulates the expression of *HR* genes by activating RNA polymerase II (POLR2A) through phosphorylation of its C-terminal domain [77,91]. It regulates gene transcription, RNA splicing, translation, cell cycle, cell proliferation, DDR and maintenance of genomic stability [92,93]. No definitive association between any hereditary cancer and *CDK12* has been described or validated in the literature. However, a case–control study which included patients of Tatar descent suggested a possible association between a variant of *CDK12* (c.1047-2A>G) and hereditary breast and ovarian cancer [77]. Somatic PVs have been described in breast, ovarian, prostate and pancreatic cancer with a frequency of 0.8%, 2.7%, 4% and 0.1%, respectively [79,80]. In ovarian cancer, clinical trial studies reported around 1,5% [35]. Preclinical studies have shown that *CDK12* PVs confer higher sensitivity of tumor cells to PARPis [50,51]. The HRD status is not associated with *CDK12* PVs [35].

***CHEK2-CHEK1***

*CHEK2* and *CHEK1* are serine/threonine-protein kinases implicated in cell-cycle arrest in response to DNA damage, interacting with several proteins in the HR pathway (*ATM*, *ATR*, *BRCA2*) [77]. No association between any hereditary cancer and *CHEK1* has been validated to date. Germline PV affecting *CHEK2* may increase the risk of breast cancer 1.5-fold [84]. Emerging evidence suggests an association with increased prostate cancer risk. Although the *CHEK2* c.1100del variant has also been proposed to be associated with colorectal cancer (CRC) [94], this association was not confirmed in a subsequent population-based study. Two meta-analysis reported a modestly increased CRC risk of about twofold [95,96]. Somatic PVs in *CHEK1* have been reported in 0.6%, 0.7% and 0.5% of breast, ovarian and prostate cancers, respectively. Somatic alterations affecting *CHEK2* have been described in 0.3%, 1%, 1.5% and 0.5% of breast, ovarian, prostate and pancreatic cancers, respectively [79,80,97]. Preclinical studies have shown that loss of function of *CHEK1* and *CHEK2* confers higher sensitivity of cancer cells to PARPis [36,46]. The association between *CHEK2* PVs and HRD status was inconsistent [35].

***FANCs***

The Fanconi anemia (FA) family includes 22 distinct functional complementation genes: *FANCA*, *FANCB*, *FANCC*, *FANCD1 (BRCA2)*, *FANCD2*, *FANCE*, *FANCF*, *FANCI*, *FANCJ (BRIP1)*, *FANCL*, *FANCM*, *FANCN (PALB2)*, *FANCO (RAD51C)*, *FANCP (SLX4)*, *FANCQ (ERCC4)*, *FANCR (RAD51)*, *FANCS (BRCA1)*, *FANCT (UBE2T)*, *FANCU (XRCC2)*, *FANCV (REV7)*. The FA pathway, also called the FA-BRCA pathway, is a fundamental DNA repair pathway that recognizes DNA damage and orchestrates DNA damage responses, especially for DNA interstrand crosslink (ICL) repair [98]. Roles in the repair of other types of DNA damage and in the regulation of replication stress responses have been additionally ascribed. One FA gene subset encodes nine proteins of the FA core complex (FANCA/B/C/E/F/G/L/M/T), which activates the FANCI–FANCD2 heterodimer through monoubiquitination. The remaining eight FA proteins (FANCD1/J/N/O/P/Q/R/S) mediate recombinational and nucleolytic reactions to complete repair [77,99]. For example, excision repair cross-complementation group 4 (FANCQ/ERCC4/XPF) is a gene that encodes a protein that is involved in nucleotide excision repair. The most common alterations in ERCC4 are *ERCC4* PV (1.37%), *ERCC4* Amplification (0.15%), *ERCC4* E527D (0.08%), *ERCC4* Loss (0.03%), and *ERCC4* S540L (0.02%). The evidence available so far suggests that ERCC4 is not a cancer susceptibility gene. *ERCC4* is altered in 1.51% of breast carcinoma patients, 0.85% of ovarian carcinoma tumor, 0.75% of prostate adenocarcinoma patients, and is altered in 0.7% of pancreatic carcinoma [100]. Of note, increased hereditary risk for cancer has been associated with some of other FA genes (*FAND1 (BRCA2)*, *FANCS (BRCA1)*, *FANCN (PALB2)*, *FANCO (RAD51C)*). Somatic PVs in the FA-BRCA pathway have been reported in around 0.3–3% of breast, ovarian, prostate and pancreatic cancers [79,80]. Preclinical studies have shown that loss of function of *FANCA*, *FANCC*, *FANCD2* and *FANCG* confers higher sensitivity of cancer cells to PARPis [36,39,52,53]. The association between *FANCA* PVs and HRD status was inconsistent [35]. The HRD status is not associated with *FANCM*, *FANCI*, or *FANCL* PVs in ovarian cancer [35].

***HDAC2***

Histone deacetylase 2 (HDAC2) is an enzyme encoded by the *HDAC2* gene. This protein is involved in chromatin remodeling through deacetylation of core histones and plays a role in transcriptional regulation and cell-cycle control. Following the identification of inactivating *HDAC2* PVs in cancer, studies demonstrated that HDAC inhibition can suppress tumor growth in colorectal cancer cell lines [101]. Although *HDAC2* is not a canonical homologous recombination (HR) gene and *HDAC2* PVs have not been validated as predictive biomarkers of PARP inhibitor response, preclinical data indicate that HDAC inhibition can induce a functional “BRCAness” phenotype by downregulating HR-related proteins such as *BRCA1* and *RAD51* [102]. More recently, dual PARP/HDAC inhibitors have been shown to restore synthetic lethality and enhance antitumor activity through combined DNA damage and immune signaling activation in triple-negative breast cancer models [103].

***MRE11***

*MRE11* has exonuclease and endonuclease activity. It is a component of the MRN complex (*MRE11-RAD50-NBN*) which is implicated in activating DNA damage sensors in the HR pathway [77,104]. No association between any hereditary cancer and this gene has been validated to date [84]. Somatic PVs have been reported in 0.5% of breast and prostate cancers, and in 0.6% and 0.1% of ovarian and pancreatic cancers, respectively [79,80]. Preclinical studies have shown that loss of function of this gene is associated with higher sensitivity to PARPis [54,55,56,57,105].

***NBN***

*NBN* is a component of the MRN complex (*MRE11-RAD50-NBN*) which is implicated in double-stranded break repair. It activates and recruits signal check-point sensors (*ATM*, *ATR*) and other proteins like *MRE11* and *RAD50* to the site of DNA damage [77,104]. Germline PVs in the *NBN* gene have been described and confer a relative risk of ovarian cancer development nearly twice that of the normal population [61,84]. Women with *NBN* PVs are also at increased risk of developing breast cancer [106]. Somatic PVs have been described in 0.1%, 0.5%, 0.3% and 0.4% of breast, ovarian, prostate and pancreatic cancers, respectively [79,80]. Preclinical studies have shown that loss of function of NBN is associated with higher sensitivity to PARPis [36,55]. *NBN* pathogenic variants were not associated with HRD status in ovarian cancer [35].

***PALB2***

*PALB2* plays a critical role in the HR pathway through different mechanisms [107]. It binds DNA with high affinity for the D loop and recruits *BRCA2* and *RAD51* to DNA break sites [108]. It promotes stability of *BRCA2* and is essential for its checkpoint functions. It also forms a complex with *BRCA1* and *BRCA2*, serving as a bridge of these two proteins, which is an essential step for HR repair [77,109]. Germline PVs in *PALB2* confer a 5-times-higher risk of breast cancer and 2-times-higher risk of ovarian cancer compared to the normal population [84]. Estimated lifetime risk is 53% for breast cancer, 5% for ovarian cancer and 2–3% for pancreatic cancer [110,111]. Somatic PVs have been reported in 0.2%, 0.5%, 0.4% and 0.2% of breast, ovarian, prostate and pancreatic cancers, respectively [79,80]. Preclinical studies have shown that loss of function of *PALB2* confers higher sensitivity of cancer cells to PARPis [53,58]. The HRD status is associated with *PALB2* PV [35].

***PPP2R2A***

Protein phosphatase 2A (PP2A) exhibits tumor suppressor function, and the reduced expression of *PPP2R2A* promotes tumor pathogenesis [112]. *PPP2R2A*, located at chromosome 8p21.2, is deleted at high frequencies in prostate, ovarian, and luminal type B breast cancers [113]. *PPP2R2A* is altered in 1.42% of breast carcinoma patients [83,100] and is associated with poor prognosis in prostate adenocarcinomas [112]. *PP2A* genes are heterozygous-lost in the majority of HGSOC (high-grade serous ovarian cancer) and this loss correlates with worse survival. Preclinical studies showed that SMAP-061-induced stabilization of PP2A inhibits the HR output by targeting RAD51, leading to chronic accumulation of DNA damage and ultimately apoptosis. This evidence emphasize the potential of PP2A modulators to overcome PARPi insensitivity, given that targeting RAD51 presents benefits in overcoming PARPi resistance driven by *BRCA1/2* mutation reversions [114]. In HGSOC, PPP2R2A is frequently deleted/downregulated, and ovarian cancer cells with low PPP2R2A expression showed increased sensitivity to the PARP inhibitor niraparib *in vitro*; however, this evidence remains limited and requires validation in larger preclinical models and clinically annotated cohorts [115].

***PTEN***

PTEN encodes a phosphatase whose role in the control of the phosphoinositide 3 kinase (PI3K) signaling pathway is well established [116]. Mutations in the phosphatase and tensin homolog (PTEN) gene and loss of PTEN expression have both been associated with a wide range of human tumors. A new functional role for PTEN was recently suggested by the observation that mouse embryonic *Pten*−/− cells exhibit genomic instability, a phenotype ascribed to either defects in RAD51-mediated DNA double-strand break repair (DSBR) [117] or defects in cell-cycle checkpoints [118]. Germline *PTEN* pathogenic variant confers a lifetime risk of 60% for breast cancers. The risk of thyroid, colorectal, endometrial, and renal cancers is also improved [119,120]. Concerning somatic alterations, TCGA data shows that PVs in *PTEN* occur across a wide range of cancers, with uterine cancer and glioblastoma multiforme having the highest percentages of *PTEN* PVs and homozygous loss. Thus far, no *in vitro* correlation has been detected between a *PTEN* mutational status and PARPi response [119,120].

***RAD50***

*RAD50* is a member of the MRN complex, essential to initiate double-stranded DNA repair. This protein plays a role in binding and holding DNA ends in close proximity [77,104]. No association between any hereditary cancer and this gene has been validated. Somatic PVs have been reported in 0.1% and 0.5% of breast and ovarian cancers, respectively, and in 0.7% of prostate and pancreatic cancers [79,80]. Preclinical studies have shown that loss of function of this gene is associated with higher sensitivity to PARPis [55,121].

***RAD51***

The RAD51 recombinase has long been considered a major guardian of genomic stability but multifaceted roles that *RAD51* plays in carcinogenesis, cancer progression and anticancer drug resistance [77,122,123]. No association between any hereditary cancer and this gene has been validated to date. Somatic PVs have been reported in 0.5%, 2.5%, 0.6% and 0.1% of breast, ovarian, prostate and pancreatic cancers, respectively [79,80]. Preclinical studies have shown that loss of function of *RAD51* confers higher sensitivity of tumor cells to PARPis [36,44,59,60].

***RAD51B/C/D/XRCC2/3***

*RAD51B/C/D* paralogs are members of the RAD51 complex (*RAD51B*, *RAD51C*, *RAD51D*, *XRCC2* and *XRCC3*) involved in HR DNA repair. They form distinct complexes that act as mediators of RAD51 presynaptic filament assembly, and they are required for loading of RAD51 at sites of DNA damage [77,124,125,126,127]. X-ray repair cross-complementing group 3 (XRCC3) is responsible for maintaining the integrity of the genome, playing a critical role in protecting it against mutations which lead to cancer [128]. Germline PVs in *RAD51C*/*D* have been associated with a four- and sevenfold higher risk of developing ovarian cancer compared to the normal population, respectively [84,129]. No association between any hereditary cancer and *RAD51B* has been validated to date. There is emerging evidence of an increased risk for breast cancer for *RAD51B* PV/VPL carriers [130,131,132]. Somatic PVs have been reported with a frequency of 0.6%, 1.2%, 0.4% and 0.2% in breast, ovarian, prostate and pancreatic cancer, respectively, for *RAD51B*; of 0.4%, 0.2%, 0.5% and 0.5% in breast, ovarian, prostate and pancreatic cancers, respectively, for *RAD51C*; and of 0.2%, 1.6%, 0.4% and 0.1% in breast and ovarian cancers, respectively, for *RAD51D* [79,80]. The genetic defects in *XRCC2* and *XRCC3* genes can affect repairing efficiency as the genes are highly polymorphic, which could lead to the progression of ovarian cancer [133].

Preclinical studies have shown that loss of function of *RAD51C* [61] and *RAD51D* [62] confers higher sensitivity of cancer cells to PARPis. The HRD status is associated with *RAD51C*, *RAD51D* PVs. The HRD status is inconsistently associated with *RAD51B* PVs [35].

***RAD52***

RAD52 was first identified along with a large group of homologous recombination proteins in a screen for DNA-repair-deficient *S. cerevisiae* mutants following ionizing radiation. Nonetheless, RAD52 is critically important for most, if not all, recombination events in yeast including meiotic recombination, homologous DNA integration, and mating-type switching [134,135]. As *RAD52* PV cause no discernible homologous recombination phenotype in humans, the synthetically lethal BRCA/RAD52 relationship makes RAD52 an attractive therapeutic target. It is important to develop more work to create truly drug-like RAD52 inhibitors that can be used in clinic [136,137]. Thus far, no *in vitro* correlation has been detected between a *RAD52* PV and PARPi response [119,120].

***RAD54L***

*RAD54L* (*RAD54L* gene) is a member of the DEAD-like helicase superfamily and plays a role in dissociating *RAD51* from nucleoprotein filaments on double-stranded DNA [124]. No association between any hereditary cancer and this gene has been validated to date, while somatic PVs affecting *RAD54L* have been reported in the literature. Conflicting preclinical data have been published concerning the association of genomic alterations in *RAD54L* with the sensitivity of tumor cells to PARPis. Some studies have reported increased sensitivity after *RAD54L* knock down whilst others have showed the opposite [36,48,53]. The HRD status is not associated with *RAD54L* PVs [35].

***WRN***

*WRN*, a member of the RECQ family of DNA helicases, plays essential roles at stalled forks to counteract replication stress, thereby ensuring genomic stability. Biallelic PVs of the *WRN* gene are associated with a rare syndrome (Werner syndrome) characterized by shot statures, signs of premature aging and an increased risk of developing several specific types of cancer, mostly thyroid epithelial neoplasms, melanoma, meningiomas, soft tissue sarcomas, leukemia and preleukemic conditions, and primary bone neoplasms. Thus far, no *in vitro* correlation has been detected between *WRN* mutations, and PARPi response has been published. In addition, genetic or pharmacologic inhibition of *WRN* selectively induces DNA damage and apoptosis in microsatellite instability-high (MSI-H) tumor cells with sparing microsatellite-stable cells, supporting *WRN* as a promising context-dependent therapeutic target, although clinical validation remains limited [138].

## 4. Clinical Trials Results

Following the literature review, 17 articles were identified reporting use of PARPis in patients with ovarian, breast, prostate and pancreatic cancers harboring PVs in homologous recombination genes other than *BRCA1/2* (Figure 3 and Table 3).

Below are descriptions of each clinical trial **by tumor type** that include study design, PARPis in test, type of primary tumor, eligibility requirements, number of patients, and primary and secondary endpoint results.

### 4.1. Ovarian Cancer Clinical Studies

**STUDY 19 Trial ((NCT00753545)**

STUDY 19 was a randomized, double-blind, placebo-controlled phase II trial published in 2012 that evaluated olaparib as maintenance therapy in patients with platinum-sensitive recurrent high-grade ovarian, fallopian tube, or primary peritoneal cancer. Eligibility criteria included completion of at least two prior lines of platinum-based chemotherapy and achievement of a complete or partial response to the most recent platinum regimen. Patients were randomized to receive olaparib 400 mg twice daily (capsule formulation) or placebo. The primary endpoint was progression-free survival (PFS), while secondary endpoints included time to progression (TTP), overall response rate (ORR), and overall survival (OS) [154].

A total of 265 patients were enrolled, with 136 assigned to olaparib and 129 to placebo. In a retrospective exploratory analysis, 21 of the 136 patients treated with olaparib were identified as harboring pathogenic variants (PVs) in homologous recombination (HR) genes other than *BRCA1/2*. These PVs included *BRIP1* (*n* = 5; 4 germline and 1 somatic), *CDK12* (*n* = 3), *RAD54L* (*n* = 3), *RAD51B* (*n* = 2), *RAD51C* (*n* = 1, germline), *RAD52* (*n* = 1), *ATM* (*n* = 1), *FANCA* (*n* = 1), *FANCL* (*n* = 1), *FANCD2* (*n* = 1, somatic), *FANCI* (*n* = 1), and *XRCC3* (*n* = 1) [154].

In this subgroup, olaparib maintenance was associated with a significant improvement in PFS, with a hazard ratio (HR) of 0.21 (95% CI, 0.04–0.86) compared with placebo. The overall survival (OS) analysis showed an HR of 0.77 (95% CI, 0.28–2.28), although this result was not statistically significant [154].

**ENGOT-OV16/NOVA Trial (NCT01847274)**

The ENGOT-OV16/NOVA was a randomized, double-blind, phase III trial evaluating niraparib as maintenance therapy in patients with platinum-sensitive recurrent high-grade serous ovarian, fallopian-tube, or primary peritoneal cancer [140]. Eligible patients had received at least two prior lines of platinum-based chemotherapy and were randomized within 8 weeks after completion of their last platinum regimen to receive niraparib (300 mg once daily) or placebo until disease progression or unacceptable toxicity. The primary endpoint was progression-free survival (PFS).

Patients were stratified according to the presence of a germline *BRCA1/2* pathogenic variant (*gBRCA* cohort) or absence thereof (non-g*BRCA* cohort), with a predefined HRD analysis performed in the non-g*BRCA* population. Among the 553 enrolled patients, 203 were classified as *gBRCA* and 350 as non-g*BRCA*. Of the 345 non-g*BRCA*, 115 were HRD-positive (without somatic PVs in *BRCA*). In the *gBRCA* cohort, niraparib significantly prolonged median PFS compared with placebo (21.0 *vs.* 5.5 months), corresponding to a hazard ratio (HR) of 0.27 (95% CI, 0.17–0.41). In the non-g*BRCA* HRD-positive subgroup, median PFS was 12.9 months with niraparib versus 3.8 months with placebo (HR 0.38; 95% CI, 0.24–0.59). In the overall non-g*BRCA* population, niraparib also conferred a significant PFS benefit (9.3 *vs.* 3.9 months; HR 0.45; 95% CI, 0.34–0.61) [140]. Niraparib significantly improved progression-free survival in **non-g*BRCA*** ovarian cancer, particularly in **HRD**-positive tumors, but the study did not report outcomes according to individual non-*BRCA HR* genes.

**ARIEL2 Trial (NCT01891344)**

The ARIEL2 trial was an open-label, phase II study that evaluated rucaparib monotherapy in patients with relapsed, platinum-sensitive high-grade ovarian cancer [25]. Eligible patients had high-grade serous or endometrioid ovarian, fallopian tube, or primary peritoneal carcinoma and had received at least one prior platinum-based chemotherapy regimen. Patients were treated with rucaparib at a dose of 600 mg twice daily until disease progression or treatment discontinuation.

The primary endpoint was progression-free survival (PFS). Secondary endpoints included objective response rate (ORR), defined as a complete or partial response according to RECIST criteria, and duration of response. Tumors were prospectively classified into three homologous recombination deficiency (HRD) subgroups based on tumor *BRCA* status and genomic loss of heterozygosity (LOH): *BRCA*-mutant, *BRCA* wild-type with high LOH (LOH-high), and *BRCA* wild-type with low LOH (LOH-low).

Among the 204 enrolled patients, all received rucaparib and were allocated to the following three subgroups: 40 patients with tumor *BRCA* pathogenic variants, 82 with LOH-high tumors, and 70 with LOH-low tumors; LOH status could not be determined in 12 *BRCA* wild-type tumors. Median PFS was 12.8 months in the *BRCA*-mutant subgroup, 5.7 months in the LOH-high subgroup, and 5.2 months in the LOH-low subgroup. Corresponding ORRs were 80%, 29%, and 10%, respectively.

An exploratory analysis was performed in *BRCA* wild-type tumors harboring pathogenic variants in other homologous recombination genes. Patients with *RAD51C* pathogenic variants (*n* = 4) demonstrated an ORR of 75% (three partial responses and one stable disease). Two patients with *ATM* pathogenic variants were included; only one had measurable disease and achieved stable disease. No objective responses were observed in patients with pathogenic variants in *BRIP1* (*n* = 2), *RAD51D* (*n* = 2), *CHEK2* (*n* = 2), *FANCM* (*n* = 2), *FANCA* (*n* = 1), *FANCI* (*n* = 1), *RAD51B* (*n* = 1), or *RAD54L* (*n* = 1). One patient with an *NBN* pathogenic variant achieved a complete response, while the second had stable disease, corresponding to an ORR of 50% in this small subgroup.

Overall, ARIEL2 demonstrated that rucaparib activity extends beyond *BRCA*-mutant tumors, particularly in *BRCA* wild-type, HRD-positive cancers. However, responses among non-*BRCA HR* gene alterations were heterogeneous and limited by small sample sizes [25]. Rucaparib showed heterogeneous activity in BRCA-wild-type tumors harboring pathogenic variants in *RAD51C*, *ATM*, *BRIP1*, *RAD51D*, *CHEK2*, *FANCM*, *FANCA*, *FANCI*, *RAD51B*, *RAD54L*, and *NBN*. The most notable responses were observed in tumors with *RAD51C* and *NBN* pathogenic variants, whereas activity was limited or absent in several other genes.

**ARIEL3 Trial (NCT01968213)**

Of the two LOH-low patients, the PFL for Rucaparib and placebo groups were 6.7 and 5.4 months, respectively (HR 0.58; (95% CI 0.40–0.85); *p* = 0.0049).

The ARIEL3 trial was a randomized, double-blind, phase III study evaluating rucaparib as maintenance therapy in patients with platinum-sensitive, recurrent high-grade ovarian cancer [141]. Eligible patients had high-grade serous or endometrioid ovarian, fallopian tube, or primary peritoneal carcinoma, had received at least two prior platinum-based chemotherapy regimens, and had achieved a complete or partial response to their most recent platinum-based doublet, which was administered for a minimum of four cycles and assessed according to RECIST criteria.

Patients were randomized in a 2:1 ratio to receive rucaparib (600 mg twice daily) or placebo until disease progression, death, or unacceptable toxicity. The primary endpoint was investigator-assessed progression-free survival (PFS), with secondary endpoints including PFS assessed by blinded independent central review (BICR).

Tumors were prospectively stratified according to homologous recombination deficiency (HRD) status based on tumor *BRCA1/2* pathogenic variant and genomic loss of heterozygosity (LOH), defining three subgroups: *BRCA*-mutant, *BRCA* wild-type with high LOH (LOH-high), and *BRCA* wild-type with low LOH (LOH-low). HRD-positive tumors were defined as either *BRCA*-mutant or *BRCA* wild-type with high LOH. The LOH-high category included PVs across a broad panel of homologous recombination-related genes.

A total of 564 patients were enrolled. In the *BRCA* wild-type HRD-positive subgroup (*n* = 158), median PFS was 9.7 months in the rucaparib group compared with 5.4 months in the placebo group (hazard ratio (HR) 0.44; 95% CI, 0.29–0.66; *p* < 0.0001). When assessed by BICR, median PFS was 11.1 months with rucaparib versus 5.6 months with placebo (HR 0.55; 95% CI, 0.35–0.89; *p* = 0.0135).

30 genes were associated with patients with HRD and LOH-high: *ATM*; *ATR*; *ATRX*; *BARD1*; *BLM*; *BRIP1*; *CHEK1*; *CHEK2*; *FANCA*; *FANCC*; *FANCD2*; *FANCE*; *FANCF*; *FANCG*; *FANCI*; *FANCL*; *FANCM*; *MRE11A*; *NBN*; *PALB2*; *RAD50*; *RAD51*; *RAD51B*; *RAD51C*; *RAD51D*; *RAD52*; *RAD54L*; and *RPA1*.

In the *BRCA* wild-type LOH-low subgroup, rucaparib also demonstrated a statistically significant but more modest PFS benefit, with median PFS of 6.7 months compared with 5.4 months in the placebo group (HR 0.58; 95% CI, 0.40–0.85; *p* = 0.0049). Overall, ARIEL3 demonstrated that rucaparib maintenance therapy provides a clinically meaningful PFS benefit not only in BRCA-mutant tumors but also in BRCA wild-type, HRD-positive ovarian cancers, while the benefit in HRD-negative tumors was limited [141]. The predictive value appeared to be driven mainly by LOH/HRD status rather than by any individual gene.

**PAOLA trial ((ENGOT-ov25/PAOLA-1, NCT02477644))**

The PAOLA-1 trial was a randomized, double-blind, phase III study evaluating the efficacy of maintenance therapy with olaparib in combination with bevacizumab in patients with newly diagnosed, advanced, high-grade ovarian, fallopian tube, or primary peritoneal cancer who had achieved a complete or partial response after first-line platinum–taxane chemotherapy plus bevacizumab [155]. Patients were eligible regardless of surgical outcome or *BRCA* pathogenic variant status.

Tumor homologous recombination deficiency (HRD) status was assessed using the *myChoice^®^ HRD Plus* assay (Myriad Genetic Laboratories), which incorporates *BRCA1/2* pathogenic variant status and a genomic instability score (GIS); a GIS ≥ 42 defines HRD positivity [155]. The primary endpoint was investigator-assessed progression-free survival (PFS), and overall survival (OS) was a key secondary endpoint.

A total of 806 patients underwent randomization: 537 were assigned to receive olaparib plus bevacizumab and 269 to receive placebo plus bevacizumab. As expected, a substantial benefit was observed in patients with *BRCA*-mutated tumors, with a median PFS of 37.2 months in the olaparib group compared with 21.7 months in the placebo group (HR 0.31; 95% CI, 0.20–0.47) [155]. Importantly, a clinically meaningful benefit was also demonstrated in patients with HRD-positive tumors without *BRCA* pathogenic variants, with a median PFS of 28.1 months versus 16.6 months, respectively (HR 0.43; 95% CI, 0.28–0.66) [155].

A subsequent overall survival analysis showed a trend toward improved OS in the HRD-positive/*BRCA*-wild-type subgroup treated with olaparib plus bevacizumab. At 5 years, 54.7% of patients in the olaparib group were alive compared with 44.2% in the placebo group (median OS not reached *vs.* 52.0 months; HR 0.71; 95% CI, 0.45–1.13), although this difference did not reach statistical significance [142].

More recently, an exploratory analysis of PAOLA-1 investigated whether PVs in non-*BRCA1/2* homologous recombination repair (HRR) genes could independently predict benefit from olaparib plus bevacizumab, irrespective of HRD status [35]. Progression-free survival was evaluated across six different non-*BRCA HRR* gene panels. Non-*BRCA HRR* pathogenic variants were not enriched in HRD-positive tumors (25/806; 3.1%) compared with HRD-negative tumors (33/806; 4.1%) or tumors with unknown HRD status (14/806; 1.7%) [35]. Moreover, most tumors harboring non-*BRCA HRR* pathogenic variants exhibited low median GIS values, although higher GIS values were observed in tumors with PVs in *BLM*, *BRIP1*, *RAD51C*, *PALB2*, and *RAD51D* [35]. Importantly, none of the evaluated non-*BRCA HRR* gene panels predicted a differential PFS benefit from maintenance olaparib plus bevacizumab compared with bevacizumab alone. The authors concluded that current non-*BRCA HRR* gene panels should not be considered a substitute for HRD assessment based on *BRCA* mutation status and genomic instability testing in newly diagnosed advanced ovarian cancer [35].

**OPINION Trial l (NCT03402841)**

The OPINION trial was a phase IIIb, single-arm study evaluating olaparib maintenance monotherapy in patients without a pathogenic germline *BRCA1/BRCA2* variant (*gBRCA*wt) who had platinum-sensitive relapsed ovarian cancer (PSROC) and had received at least two prior lines of platinum-based chemotherapy [143]. Patients with somatic *BRCA* pathogenic variants (*sBRCA*m) were eligible for enrollment. Retrospective centralized tumor testing was performed in all enrolled patients to assess *sBRCA* pathogenic variant status and homologous recombination deficiency (HRD) using the *myChoice^®^ HRD Plus* assay [143].

The primary endpoint was progression-free survival (PFS). A key secondary endpoint was PFS according to HRD status and *sBRCA* pathogenic variant status. A total of 279 patients were enrolled and received olaparib.

Median PFS was 16.4 months in patients with *sBRCA*m, 11.1 months in the overall HRD-positive population (including *sBRCA*m), 9.7 months in HRD-positive patients excluding *sBRCA*m, and 7.3 months in HRD-negative patients, respectively [143]. These results indicate that olaparib maintenance therapy provides clinical benefit in patients without a germline *BRCA* pathogenic variant, with the greatest benefit observed in tumors harboring *sBRCA* mutations and in HRD-positive disease, although activity was also observed in HRD-negative tumors [143]. Because efficacy was not reported by individual non-*BRCA HR* genes, the study supports HRD status rather than specific gene alterations.

**LIGHT Trial (NCT02983799)**

This was a phase II, multicohort study (2022) that evaluated olaparib monotherapy in patients with platinum-sensitive relapsed ovarian cancer (PSROC) who had received ≥1 prior line of platinum-based chemotherapy [156]. Patients were prospectively assigned to four cohorts according to tumor genomic status: germline *BRCA1/2* pathogenic variants (*gBRCAm*), somatic *BRCA1/2* pathogenic variants (*sBRCAm*), HRD-positive tumors without *BRCA* pathogenic variants, or HRD-negative tumors.

The primary endpoint was objective response rate (ORR). Secondary endpoints included disease control rate (DCR) and PFS. Tumor molecular characterization was performed using the Myriad BRCAAnalysis CDx and myChoice^®^ HRD assays; HRD positivity was defined by a genomic instability score ≥ 42 [156].

Of the 272 patients enrolled, 271 received olaparib, and 270 were included in the efficacy analysis. The ORRs in the *gBRCAm*, *sBRCAm*, HRD-positive (*BRCA* wild-type), and HRD-negative cohorts were 69.3%, 64.0%, 29.4%, and 10.1%, respectively. Corresponding DCRs were 96.0%, 100.0%, 79.4%, and 75.3%. Median PFS was 11.0 months in the *gBRCAm* cohort, 10.8 months in the *sBRCAm* cohort, 7.2 months in the HRD-positive cohort, and 5.4 months in the HRD-negative cohort [156]. In the final overall survival analysis, OS rates remained highest in the germline and somatic BRCA cohorts, and among BRCA–wild-type tumors, outcomes continued to favor HRD-positive over HRD-negative disease, supporting the clinical relevance of HRD beyond BRCA alterations [144].

Overall, the LIGHT trial demonstrated that olaparib has clinical activity across all biomarker-defined cohorts, with the greatest efficacy observed in patients harboring *BRCA* pathogenic variants, irrespective of germline or somatic origin. Among patients without *BRCA* PVs, improved outcomes were observed in those with HRD-positive tumors compared with HRD-negative disease, supporting the biological relevance of HRD beyond *BRCA* alterations [144,156]. As individual non-*BRCA HR* genes were not analyzed separately, the study again favors HRD positivity over gene-level interpretation.

### 4.2. Breast Cancer Clinical Studies

**TBRC-048 (NCT03344965)**

The TBCRC 048 trial was a phase II, multicenter, single-arm study that evaluated olaparib monotherapy in patients with metastatic breast cancer harboring either germline pathogenic variants (PVs) in non-*BRCA1/2* homologous recombination-related genes or somatic pathogenic variants in *BRCA1/2* or other HR-related genes [145]. Eligible patients had received up to two prior lines of chemotherapy for metastatic disease and were excluded if they had platinum-refractory disease. Olaparib was administered at a dose of 300 mg twice daily until disease progression or unacceptable toxicity.

Patients were assigned to two cohorts. Cohort 1 included patients with germline PVs in non-*BRCA HR* genes (*ATM*, *ATR*, *BAP1*, *BARD1*, *BLM*, *BRIP1*, *CHEK1*, *CHEK2*, *CDK12*, *FANCA*, *FANCC*, *FANCD2*, *FANCF*, *MRE11A*, *NBN*, *PALB2*, *RAD50*, *RAD51C*, *RAD51D*, or *WRN*), whereas cohort 2 included patients with somatic *BRCA1/2* pathogenic variants. The primary endpoint was ORR according to RECIST v1.1. Secondary endpoints included PFS, clinical benefit rate (CBR; defined as complete or partial response or stable disease ≥18 weeks), duration of response, and safety.

In cohort 1 (*n* = 27), the distribution of HR gene PVs was as follows: *PALB2* (*n* = 10), *CHEK2* (*n* = 8), *ATM* (*n* = 4), *BARD1* (*n* = 1), *RAD50* (*n* = 1), dual *CHEK2/ATM* (*n* = 2), and dual *PALB2/ATM* (*n* = 1). In this cohort, the ORR was 33%, the clinical benefit rate was 50%, the median duration of response was 9 months, and the median PFS was 4.5 months.

Gene-level exploratory analyses demonstrated marked heterogeneity in response. Among *CHEK2* carriers, no objective responses were observed, with best responses limited to stable disease. Patients with *ATM* PVs showed minimal activity, with three cases of progressive disease and one stable disease. The single patient with a *BARD1* pathogenic variant achieved prolonged stable disease (>6 months). Two patients harboring concurrent *CHEK2* and *ATM* alterations experienced disease progression. In contrast, the patient with combined *PALB2* and *ATM* PVs achieved a partial response.

Notably, patients with germline *PALB2* pathogenic variants demonstrated substantially greater benefit, with an ORR of 82%, 100% clinical benefit rate, median duration of response of 9 months, and median PFS of 13.3 months [145]. An updated analysis of an expanded cohort including 24 patients with germline *PALB2* pathogenic variants reported a confirmed ORR of 75% and a median duration of response of 12.4 months, reinforcing *PALB2* as a strong predictive biomarker of PARP inhibitor sensitivity in metastatic breast cancer [157]. Olaparib demonstrated meaningful activity in metastatic breast cancer with germline or somatic alterations in non-*BRCA HR*-related genes, especially *PALB2*, while limited or no responses were observed with *ATM* and *CHEK2*; other genes represented included *BARD1* and *RAD50*. Among *non-BRCA* genes, *PALB2* emerged as the strongest predictor of PARP inhibitor sensitivity.

**GRUBER ET AL. (NCT02401347)**

Gruber et al. conducted a phase II, open-label study evaluating talazoparib monotherapy in patients with advanced solid tumors harboring homologous recombination deficiency (HRD)-associated gene alterations other than *BRCA1/2* [146]. Patients were allocated to two cohorts: cohort A, comprising patients with triple-negative breast cancer (TNBC) with high HRD scores, and cohort B, including patients with any solid tumor carrying germline or somatic pathogenic variants in HRD-associated genes other than *BRCA1/2*.

Eligible patients had metastatic or recurrent HER2-negative breast cancer or another advanced solid tumor, with disease progression after at least one prior line of systemic therapy for advanced disease; there was no upper limit on the number of previous treatment lines. The primary endpoint was objective response rate (ORR) according to RECIST v1.1, and secondary endpoints included clinical benefit rate (CBR), progression-free survival (PFS), and safety [146].

A total of 20 patients were enrolled. Thirteen patients had HER2-negative breast cancer (eleven hormone receptor–positive and two triple-negative), and seven patients had other tumor types, including pancreatic, colorectal, mixed Müllerian uterine, testicular, and parotid acinic cell carcinomas [146].

In cohort A, responses were modest and heterogeneous. Among patients with germline *PALB2* pathogenic variants (*n* = 5), no objective responses were observed; four patients achieved stable disease and one had progressive disease as best response. Patients with somatic *PTEN* alterations (*n* = 3) showed limited benefit, with two cases of progressive disease and one stable disease. Single patients harboring somatic *ATR* or *ATM* alterations and germline *BRIP1* pathogenic variants achieved stable disease, whereas the patient with a germline *CHEK2* pathogenic variant had progressive disease. One patient carrying multiple HRD-associated alterations (*CHEK2*, *FANCA*, and *PTEN*) achieved stable disease as best response. The median PFS in cohort A was 5.6 months [146].

Overall, this study demonstrated limited clinical activity of talazoparib monotherapy in breast cancers harboring non-*BRCA* HRD-associated gene alterations, reinforcing that not all HR gene pathogenic variants confer PARP inhibitor sensitivity and highlighting the need for more refined biomarkers beyond HRD gene panels [146].

### 4.3. Pancreatic Cancer Clinical Studies

**REISS ET AL**. **(NCT03140670)**

Reiss et al. conducted a phase II, single-arm study evaluating rucaparib monotherapy in patients with locally advanced or metastatic pancreatic cancer harboring a germline or somatic pathogenic variant in *BRCA1* or *BRCA2*, or a somatic pathogenic variant in *PALB2* [147]. Eligible patients were required to have received at least 16 weeks of prior platinum-based chemotherapy without evidence of platinum-resistant disease. Rucaparib was administered at 600 mg twice daily in continuous 28-day cycles until disease progression or unacceptable toxicity. The primary endpoint was PFS, and secondary endpoints included ORR and safety [147].

A total of 46 patients were enrolled, of whom 42 were evaluable for efficacy. Among these, six patients carried germline pathogenic variants in *PALB2*. In this subgroup, the median PFS was 14.0 months (95% CI, 0.7–28.3), and the ORR was 50%, including one complete response, two partial responses, and three cases of stable disease as best response. Notably, the complete response occurred in a patient with pancreatic squamous cell carcinoma [147].

Overall, this study provided clinical evidence supporting the activity of PARP inhibition in *PALB2*-mutated pancreatic cancer, extending the benefit of PARP inhibitors beyond *BRCA1/2*-mutated disease in carefully selected, platinum-sensitive patients [147]. Rucaparib demonstrated encouraging activity in platinum-sensitive pancreatic cancer with *PALB2* pathogenic variants, including durable responses in a small subgroup. These findings provide strong clinical support for *PALB2* as a non-BRCA predictor of PARP inhibitor benefit in pancreatic cancer.

**SWOG S1513 Trial SWOG S1513 Trial (NCT02890355)**

The SWOG S1513 was a randomized phase II trial that evaluated the addition of the PARP inhibitor veliparib to chemotherapy as second-line treatment for patients with metastatic pancreatic ductal adenocarcinoma (PDAC) [148]. Prior exposure to PARP inhibitors was not allowed. Eligible patients were required to have either disease progression after one prior line of systemic therapy for metastatic PDAC or progression to metastatic disease within 3 months after completion of gemcitabine plus nab-paclitaxel administered for localized disease.

Patients were randomized to receive either veliparib plus modified FOLFIRI (mFOLFIRI) or FOLFIRI alone. In the experimental arm, veliparib was administered at 200 mg twice daily on days 1–7 of each 14-day cycle, in combination with mFOLFIRI (irinotecan 180 mg/m^2^, folinic acid 400 mg/m^2^, and continuous-infusion 5-fluorouracil [5-FU] 2400 mg/m^2^, without bolus). The control arm received standard FOLFIRI in 14-day cycles, consisting of irinotecan 180 mg/m^2^, folinic acid 400 mg/m^2^, 5-FU bolus 400 mg/m^2^, followed by 5-FU infusion 2400 mg/m^2^. The primary endpoint was OS, while secondary endpoints included ORR, PFS, and safety [148].

Patients were stratified according to molecular PVs identified by BROCA-HR sequencing into four predefined categories: *BRCA1/2*; core non-*BRCA1/2* HR genes (*PALB2*, *ATM*, *RAD51C/D*, *BRIP1*, *BARD1*); non-core DNA repair genes (*FANCA–M*, *CDK12*, *CHEK2*, *BLM*, *SLX4*, *ERCC1/4*); and wild-type or non-HR DDR genes, including *EZH2*, *MSH2*, *MSH6*, *POLD1*, *POLE*, *RIF1*, and *WRN* [148].

Among 117 enrolled patients, 115 (59 in the veliparib arm and 56 in the control arm) underwent successful BROCA-HR testing. In the veliparib arm, nine patients harbored non-*BRCA* homologous recombination-related PVs. Clinical outcomes in this subgroup were heterogeneous: one patient with a germline *ATM* pathogenic variant experienced disease progression as best response, one patient with a somatic *FANCI* PV achieved a partial response, one patient with a somatic *CDK12* PV had progressive disease, and one patient with a somatic *ERCC4* alteration achieved stable disease. Two patients with *BLM* PVs (one germline and one somatic) experienced disease progression. Additionally, one patient with dual alterations (*FANCC* germline plus *BLM* somatic) achieved stable disease, whereas another patient with *FANCM* germline and *BLM* somatic PVs had progressive disease. A patient harboring a germline *ERCC3* PV and a somatic *SLX4* PV also experienced disease progression as best response [148].

Overall, the SWOG S1513 trial did not demonstrate a survival benefit with the addition of veliparib to FOLFIRI in an unselected metastatic PDAC population. Moreover, exploratory molecular analyses did not identify a clear subgroup of patients with non-*BRCA* homologous recombination gene alterations who derived consistent clinical benefit from veliparib, highlighting the challenges of translating preclinical PARP inhibitor sensitivity into meaningful clinical outcomes in pancreatic cancer [148].

In metastatic pancreatic cancer, multiple genes have been evaluated in tumors with non BRCA HR or DDR alterations, including *ATM*, *FANCI*, *CDK12*, *ERCC4*, *BLM*, *FANCC*, *FANCM*, *ERCC3*, and *SLX4*. Exploratory analyses did not identify any *non-BRCA* gene subgroup with consistent clinical benefit.

### 4.4. Prostate Cancer Clinical Studies

**TOPARP B Trial (NCT01682772)**

The TOPARP-B trial was a multicenter, randomized phase II study that evaluated olaparib in patients with metastatic castration-resistant prostate cancer (mCRPC) harboring DNA damage repair (DDR) gene aberrations [76]. Eligible patients were required to have received at least one and no more than two prior taxane-based chemotherapy regimens, irrespective of previous exposure to novel hormonal agents. Prior treatment with PARP inhibitors, mitoxantrone, cyclophosphamide, or platinum-based chemotherapy was not permitted.

Patients were randomized to receive olaparib 300 mg twice daily (bid) or 400 mg bid, administered continuously until disease progression, unacceptable toxicity, or treatment discontinuation at patient request. Patients initially assigned to 300 mg bid were allowed to escalate to 400 mg bid after confirmed radiographic progression [76].

The primary endpoint was confirmed response, defined as a composite of at least one of the following:

(i) Radiological objective response according to RECIST criteria;
(ii) A ≥50% decline in prostate-specific antigen (PSA50);
(iii) Conversion of circulating tumor cell count to <5 cells per 7.5 mL of blood.

Secondary endpoints included radiographic progression-free survival (rPFS), time to radiographic progression, disease-free survival, and overall survival [76].

Patients were categorized into five non–mutually exclusive DDR gene subgroups based on PVs: *BRCA1/2*, *ATM*, *CDK12*, *PALB2*, and Other DDR genes (genes potentially associated with DNA repair deficiency or PARP inhibitor sensitivity). Patients harboring PVs in more than one gene were included in all relevant subgroup analyses.

Among 592 patients with evaluable tumor tissue, 161 (27%) harbored DDR gene aberrations. Of these, 92 patients were evaluable for the primary endpoint analysis. Twenty-five patients had PVs in *BRCA1/2* and showed the highest ORR, consistent with prior TOPARP-A findings [76].

In the non-*BRCA* subgroups, clinical activity varied by gene. Patients with *ATM* PVs (*n* = 21) achieved an ORR of 36.8% and a median rPFS of 5.8 months. Those with *CDK12* PVs (*n* = 7) had an ORR of 25% and a median rPFS of 2.9 months. Patients with *PALB2* PVs (*n* = 7) demonstrated a notably higher activity, with an ORR of 57.1% and a median rPFS of 5.3 months. The heterogeneous “Other DDR” gene subgroup (*n* = 20) had a more modest benefit, with an ORR of 20% and a median rPFS of 2.8 months [76].

The “Other DDR” subgroup included PVs in the following genes: *WRN* (*n* = 7), *FANCA* (*n* = 5), *CHEK2* (*n* = 5), *FANCF* (*n* = 2), *FANCM* (*n* = 2), *ARID1A* (*n* = 1), *ATRX* (*n* = 1), *FANCG* (*n* = 1), *FANCI* (*n* = 1), *CHEK1* (*n* = 1), *RAD50* (*n* = 1), *MSH2* (*n* = 1), and *NBN* (*n* = 1). Responses within this subgroup were inconsistent, underscoring the biological heterogeneity of non-*BRCA* DDR alterations [76].

Overall, TOPARP-B confirmed the robust efficacy of olaparib in *BRCA1/2*-altered mCRPC and demonstrated variable but clinically relevant activity in selected non-*BRCA* DDR gene alterations, particularly *PALB2*. In contrast, responses associated with *ATM*, *CDK12*, and Other DDR genes were less consistent, highlighting the need for refined biomarkers beyond gene panels alone to predict PARP inhibitor benefit in prostate cancer [76].

Olaparib showed variable activity across non-BRCA DDR genes in mCRPC, with the most promising results in *PALB2*, more modest benefit in *ATM* and *CDK12*, and heterogeneous activity in other genes including *WRN*, *FANCA*, *CHEK2*, *FANCF*, *FANCM*, *ARID1A*, *ATRX*, *FANCG*, *FANCI*, *CHEK1*, *RAD50*, *MSH2*, and *NBN*. These findings suggest that only selected *non-BRCA* PVs may predict sensitivity to PARP inhibition in pancreatic cancers.

**TRITON-2 Trial (NCT02952534)**

The TRITON2 trial was a multicenter, open-label phase II study that evaluated rucaparib in patients with metastatic castration-resistant prostate cancer (mCRPC) harboring pathogenic non-*BRCA* DDR gene alterations [158]. Eligible patients were required to have received at least one prior taxane-based chemotherapy regimen and one to two lines of next-generation androgen receptor (AR)-directed therapy. Patients previously treated with PARP inhibitors, mitoxantrone, cyclophosphamide, or platinum-based chemotherapy were excluded.

Patients received rucaparib 600 mg twice daily (bid) until disease progression, death, or treatment discontinuation.

The primary endpoints were radiographic objective response rate (rORR)—defined as complete or partial response per RECIST criteria—and PSA response rate, defined as a ≥50% decline from baseline (PSA50) confirmed by a second consecutive measurement at least three weeks later. Secondary endpoints included clinical benefit rate, time to PSA progression (PSA TTP), and safety [158].

A total of 78 patients with non-*BRCA* DDR PVs were included and grouped into four non–mutually exclusive gene cohorts: *ATM*, *CDK12*, *CHEK2*, and Other DDR genes, which included *BARD1*, *BRIP1*, *FANCA*, *NBN*, *PALB2*, *RAD51*, *RAD51B*, *RAD51C*, *RAD51D*, and *RAD54L*. Patients with co-occurring PVs were included in multiple cohorts.

In the *ATM* cohort (*n* = 49), 19 patients had measurable disease, with an rORR of 10.5%, including 2 partial responses, 9 cases of stable disease, and 7 cases of progressive disease; one patient was non-evaluable. The PSA response rate was 4.1% (4/49), the median PSA TTP was 3.1 months, and the clinical benefit rate at 6 and 12 months was 28.6% and 16.7%, respectively [158].

In the *CDK12* cohort (*n* = 15), 10 patients had measurable disease, with no radiographic responses observed (rORR 0%). Best responses included six cases of stable disease and three of progressive disease, with one non-evaluable patient. The PSA response rate was 6.7%, median PSA TTP was 3.2 months, and the clinical benefit rate at 6 and 12 months was 20% and 7.1%, respectively [158].

In the *CHEK2* cohort (*n* = 12), nine patients had measurable disease, with an rORR of 11.1%, corresponding to one partial response, alongside six cases of stable disease and two of progressive disease. The PSA response rate was 16.7%, the median PSA TTP was 7.4 months, and the clinical benefit rate at 6 months was 37.5%, decreasing to 0% at 12 months [158].

The Other DDR gene cohort (*n* = 14) demonstrated the most favorable outcomes among non-*BRCA* groups. All patients had measurable disease, with an rORR of 28.6%, including three partial responses and one complete response, eight cases of stable disease, and one case of progressive disease; one patient was non-evaluable. The PSA response rate was 35.7%, the median PSA TTP was 11.1 months, and the clinical benefit rate at 6 and 12 months was 54.5% and 37.5%, respectively. This cohort included alterations in *FANCA* (*n* = 4), *NBN* (*n* = 4), *BRIP1* (*n* = 2), *PALB2* (*n* = 2), *RAD51* (*n* = 1), and *RAD51B/RAD54L* (*n* = 1) [158].

Overall, TRITON2 demonstrated limited activity of rucaparib in mCRPC patients with isolated *ATM*, *CDK12*, or *CHEK2* alterations, while more meaningful responses were observed in a subset of patients with other non-*BRCA* DDR gene alterations, particularly those involving *PALB2*, *FANCA*, and *NBN*. These findings highlight the heterogeneity of PARP inhibitor sensitivity across non-*BRCA* DDR alterations and underscore the need for refined molecular stratification beyond single-gene categorization [158].

In this assay, rucaparib showed limited activity in patients with *ATM*, *CDK12*, and *CHEK2* alterations, whereas some responses were observed in the more heterogeneous “Other DDR genes” group, including *PALB2*, *FANCA*, *NBN*, *BRIP1*, *RAD51*, *RAD51B*, and *RAD54L*. This trial highlights the strong heterogeneity of PARP inhibitor sensitivity across non-BRCA genes.

**PROfound Trial (NCT02987543)**

The PROfound trial was a randomized, open-label, phase III study that evaluated olaparib in patients with metastatic castration-resistant prostate cancer (mCRPC) harboring alterations in DNA damage repair (DDR) genes, whose disease had progressed during prior treatment with a novel androgen receptor-targeted agent (enzalutamide or abiraterone) [75].

Eligible patients were randomized in a 2:1 ratio to receive either olaparib 300 mg twice daily (bid) until disease progression or unacceptable toxicity, or physician’s choice of hormonal therapy, consisting of enzalutamide (160 mg once daily) or abiraterone (1000 mg once daily plus prednisone 5 mg bid).

Patients were stratified into two predefined cohorts based on tumor genomic profiling:

- Cohort A included patients with alterations (PVs) in *BRCA1*, *BRCA2*, or *ATM*, regardless of co-occurring PVs in other genes.
- Cohort B included patients with PVs in any of 12 other prespecified DDR genes: *BRIP1*, *BARD1*, *CDK12*, *CHEK1*, *CHEK2*, *FANCL*, *PALB2*, *PPP2R2A*, *RAD51B*, *RAD51C*, *RAD51D*, or *RAD54L*.

The primary endpoint was radiographic progression-free survival (rPFS), assessed by blinded independent central review (BICR). Secondary endpoints included ORR, time to pain progression, and overall survival (OS) [75].

A total of 387 patients were enrolled; 245 were assigned to Cohort A and 142 to Cohort B. Importantly, PROfound was not powered to detect gene-specific effects within Cohort B, and analyses for individual non-*BRCA* genes were exploratory.

In Cohort A, olaparib significantly improved radiographic progression-free survival compared with control (median 7.4 *vs.* 3.6 months; HR 0.34), as well as overall survival (19.1 *vs.* 14.7 months; HR 0.69), with a markedly higher objective response rate (~33% *vs.* ~2%).

In Cohort B, the median rPFS was 5.6 months in the olaparib arm versus 3.5 months in the control arm, with an HR of 0.88 by BICR, indicating no statistically significant benefit. Median overall survival in Cohort B was 14.2 months in the olaparib group versus 11.5 months in the control group (HR 0.73) [75].

Exploratory subgroup analyses showed heterogeneous outcomes across individual *non-BRCA DDR* genes. Patients with *ATM* PVs had a median rPFS of 5.36 months with olaparib (HR 1.04) and a median OS of 17.3 months (HR 0.82), indicating minimal benefit. Patients with *CDK12* PVs had a median rPFS of 5.1 months (HR 0.74) and a median OS of 14.2 months (HR 0.65). For other individual genes, rPFS by BICR was 5.59 months for *CHEK2*, 2.69 months for *PPP2R2A*, 10.89 months for *RAD51B*, and 7.2 months for *RAD54L*. However, patient numbers were very small, precluding definitive conclusions [75].

Overall, the PROfound trial demonstrated a clear clinical benefit of olaparib in mCRPC patients with *BRCA1/2* PVs, while patients with non-*BRCA* DDR gene PVs—particularly *ATM* and *CDK12*—derived limited or no benefit. These findings underscore that not all DDR gene PVs confer PARP inhibitor sensitivity, highlighting the need for refined biomarker strategies beyond inclusion in broad DDR gene panels [75].

In Cohort B of PROfound, olaparib did not show a clear overall benefit across *non-BRCA DDR* genes, including *BRIP1*, *BARD1*, *CDK12*, *CHEK1*, *CHEK2*, *FANCL*, *PALB2*, *PPP2R2A*, *RAD51B*, *RAD51C*, *RAD51D*, and *RAD54L*. Exploratory analyses suggested that inclusion in a broad DDR panel alone is insufficient to predict PARP inhibitor benefit.

**TALAPRO-1 Trial (NCT03148795)**

The TALAPRO-1 trial was a multicenter, open-label, phase II study that evaluated talazoparib monotherapy in patients with metastatic castration-resistant prostate cancer (mCRPC) harboring alterations in DDR genes involved in HRR [150].

Eligible patients had evidence of progressive disease, defined by at least one of the following: a minimum of three rising PSA values obtained at least one week apart, soft-tissue progression according to RECIST, or bone disease progression defined by the appearance of two or more new metastatic lesions on bone scan. Patients were required to have received one to two prior systemic chemotherapy regimens (including at least one taxane) in either the castration-sensitive or castration-resistant setting.

Enrollment was restricted to patients with pathogenic alterations in one of the following 11 DDR/HRR genes: *ATM*, *ATR*, *BRCA1*, *BRCA2*, *CHEK2*, *FANCA*, *MLH1*, *MRE11A*, *NBN*, *PALB2*, or *RAD51C*. Patients received talazoparib 1 mg once daily until disease progression or unacceptable toxicity.

The primary endpoint was ORR, defined as complete or partial response according to RECIST in patients with measurable disease. Secondary endpoints included radiographic progression-free survival (rPFS), OS, and PSA time to progression (PSA-TTP) [150].

A total of 127 patients were enrolled, of whom 104 had measurable disease and were included in the efficacy analysis. Among these, 43 patients harbored non-*BRCA* DDR alterations, including 17 with *ATM* alterations, 4 with *PALB2* alterations, and 22 with alterations in Other DDR genes (*ATR*, *CHEK2*, *FANCA*, *MLH1*, *MRE11A*, *NBN*, or *RAD51C*).

In the *ATM* subgroup (*n* = 17), best responses included six cases of stable disease, eight cases of progressive disease, one partial response, one complete response, and one non-evaluable patient, resulting in an ORR of 12%. Median rPFS was 3.5 months, OS was 12.2 months, and PSA-TTP was 3.7 months [150].

In the *PALB2* subgroup (*n* = 4), responses included two cases of stable disease, one partial response, and one non-evaluable patient, yielding an ORR of 25%. Median rPFS was 5.6 months, OS was 16.0 months, and PSA-TTP was 5.5 months [150]. In the Other DDR gene subgroup (*n* = 22), which included *ATR*, *CHEK2*, *FANCA*, *MLH1*, *MRE11A*, *NBN*, and *RAD51C*, no objective responses were observed (ORR 0%). Median rPFS was 1.8 months, OS was 10.1 months, and PSA-TTP was 3.7 months [150].

Overall, the TALAPRO-1 trial demonstrated that talazoparib has meaningful antitumor activity primarily in mCRPC patients with *BRCA1/2* alterations, while clinical benefit in non-*BRCA* DDR gene alterations—particularly *ATM* and other HRR genes—was limited and heterogeneous. These findings further support the concept that not all HRR gene alterations confer equivalent sensitivity to PARP inhibition in prostate cancer, reinforcing the need for gene-specific and functional biomarkers [150]. Talazoparib showed limited activity in mCRPC with non-BRCA DDR alterations, with modest signal in *PALB2* and little benefit in most other genes, including *ATM*, *ATR*, *CHEK2*, *FANCA*, *MLH1*, *MRE11A*, *NBN*, and *RAD51C*. These results support a selective, gene-specific approach rather than broad inclusion of all HRR genes.

**GALAHAD Trial (NCT02854436)**

Phase II study (2022) evaluated Niraparib in mCRPC with DNA repair gene defects (DRD) [151]. Eligibility included disease progression on an androgen signaling inhibitor and taxane chemotherapy, as well as a deleterious germline or somatic alteration found in at least one of the following genes: *ATM*, *BRCA1*, *BRCA2*, *BRIP1*, *CHEK2*, *FANCA*, *HDAC2*, and *PALB2*. Patients received niraparib 300 mg once daily until treatment discontinuation, death, or study termination.

The two cohorts were based on germline pathogenic or biallelic pathogenic alterations in *BRCA1* or *BRCA2* (BRCA cohort) and other prespecified non-BRCA genes (non-BRCA cohort). The primary endpoint was ORR (partial or complete response) in the measurable BRCA cohort. Secondary endpoints were OS, rPFS, and PSA TTP, and ORR in the non-BRCA cohort.

The *Non-BRCA* cohort had 81 patients; 12 had mutations in multiple genes, while the remaining 69 had single gene PVs as follows: 37 *ATM*, 18 *FANCA*, 8 *HDAC2*, 5 *CHECK2*, and 1 *BRIP1*. Of the 81 patients, 47 had measurable disease.

Secondary endpoints for total non-*BRCA* (*n* = 69) were 9.63-month (8.05–13.44) OS, 3.71-month (1.97–5.49) rPFS, and 3.65-month (2.83–3.71) PSA TTP. Among the measurable non-*BRCA* cohort (*n* = 81), the ORR was 10.6% (five patients) with no complete responses.

Niraparib showed low response rates in the non-BRCA cohort, which included *ATM*, *FANCA*, *HDAC2*, *CHEK2*, and *BRIP1*, suggesting that these alterations as a group are weak predictors of PARP inhibitor benefit in prostate cancer. Clinical activity was clearly lower than that observed in *BRCA1/2*-altered disease.

**PROpel study (NCT03732820)**

The PROpel trial was a randomized, double-blind, phase III study that evaluated first-line treatment with abiraterone plus prednisone/prednisolone combined with either olaparib (300 mg orally twice daily) or placebo in patients with metastatic castration-resistant prostate cancer (mCRPC), unselected for HRRm status [152,153]. Eligible patients had not received prior treatment for mCRPC and were allowed regardless of prior therapies in the castration-sensitive setting.

Patients were prospectively classified into three biomarker subgroups based on tumor tissue and/or circulating tumor DNA (ctDNA) testing: HRRm, non-HRRm, and HRRm-unknown. The HRRm subgroup included patients with at least one pathogenic variant detected in a predefined panel of homologous recombination repair genes, while the non-HRRm subgroup comprised patients without detectable HRR gene pathogenic variants. BRCA-mutated subgroups (*BRCA1* and/or *BRCA2*) were analyzed both within the HRRm population and as a distinct prespecified subgroup.

The primary endpoint was rPFS assessed by investigator review. Overall survival (OS) was a key secondary endpoint, along with safety and exploratory biomarker analyses.

In biomarker subgroup analyses, abiraterone plus olaparib consistently favored improved rPFS compared with abiraterone plus placebo, with a greater magnitude of benefit observed in patients harboring HRR pathogenic variants, particularly *BRCA1/2*. In the HRRm subgroup, the hazard ratio (HR) for rPFS was 0.50 (95% CI, 0.34–0.73), whereas in the non-HRRm subgroup, the HR was 0.76 (95% CI, 0.60–0.97), indicating benefit across biomarker-defined populations, albeit less pronounced in HRR-wild-type tumors [152].

After a median follow-up of approximately 36 months, overall survival did not reach statistical significance between treatment arms. Median OS was 42.1 months in the olaparib plus abiraterone group versus 34.7 months in the placebo plus abiraterone group (HR 0.81; 95% CI, 0.67–1.00; *p* = 0.054) [153].

Overall, the PROpel trial demonstrated that upfront combination therapy with abiraterone and olaparib improves rPFS in unselected mCRPC patients, with the greatest benefit observed in HRRm and *BRCA*-mutated tumors, while also showing a modest but consistent benefit in non-HRRm patients. These findings support the biological rationale for PARP inhibition beyond *BRCA* alterations, although the lack of a statistically significant OS benefit highlights the need for further refinement of biomarker-driven patient selection.

Olaparib plus abiraterone improved radiographic progression-free survival in mCRPC regardless of HRR status, with greater benefit observed in HRR-mutated tumors. However, as the study did not provide robust gene-specific analyses for non-BRCA alterations, definitive conclusions for individual genes beyond *BRCA1/2* cannot be drawn.

## 5. Discussion

Clinical trials suggest that the benefit of PARPis is not limited to *BRCA*-mutated tumors but also extends to the *BRCA* wild-type group with an HRD phenotype. Different genomic markers have been suggested as potential biomarkers of sensitivity to PARPis including mutational signatures able to detect HRD [159,160,161], as well as genomic scores based on available tests (academic or commercial). However, to our knowledge, there is still no standard clinically applicable approach that can establish unequivocally whether the HR pathway is deficient or not, allowing for a binary phenotypic classification (similar to PCR-MSI which is a surrogate for DNA mismatch repair deficiency).

This review synthesizes current evidence regarding PARPi sensitivity associated with selected non-*BRCA HR* genes across different cancer types. Available data from published preclinical and clinical studies, together with ongoing clinical trials, were reviewed up to 2025. As shown in Table 2, defects affecting most of the listed genes were shown to increase cancer cell sensitivity to PARPis in the preclinical setting, including both *in vitro* and *in vivo* models. The choice of the genes are wide and are not strictly related to the HR gene core pathways. For instance, *ATM* is in a related pathway to the HR, whereas *RAD51C* is a core HR gene.

For example, a recent meta-analysis including 13 trials, published by Yi et al. [162], investigated the role of genomic methods in predicting the sensitivity of ovarian and non-ovarian cancer to PARPis. Patients were classified into two subgroups: HRD (*n* = 1175) and non-HRD (*n* = 1417). Patients classified as HRD had one of the following characteristics: germline or somatic *BRCA1/2* PVs (*n* = 697), high HRD or HRD-LOH scores (*n* = 294), low *ATM* (*n* = 125), high-level MSI (*n* = 13), loss of PTEN (*n* = 30) or aberrations in other HR DNA repair genes (*n* = 16). Patients classified as non-HRD (*n* = 1417) did not have any of the aforementioned characteristics. Pooled analysis showed that both subgroups derived some benefit from PARPi which was more pronounced in patients with *BRCA* PVs, followed by those with *BRCA1/2*wt in the HRD subgroup and finally by non-HRD patients. However, only 16 patients with aberrations in non-*BRCA HR* repair genes were included in this meta-analysis, and all were derived from the study of Mateo et al. [163]. This could have under- or overestimated the significance of PARPi efficacy in this patient category. On the other hand, Kondroshova et al. reported several cases of ovarian cancer patients with *RAD51C* and *RAD51D* alterations in whom resistance to rucaparib was due to the acquisition of secondary PV at the same gene level, capable of restoring the activity of the affected protein [164]. This finding is proof of concept that non-*BRCA HR* gene defects could affect tumor sensitivity to PARPis. However, not all gene defects have the same impact on DNA repair, nor do they have potentially the same impact across different cancer types [159].

With regard to clinical data, this review suggests that patients with tumors harboring aberrations in some non-*BRCA* homologous recombination genes may derive some benefit from PARP inhibitors. However, the magnitude of benefit appears to be lower than that observed in *BRCA1/2*-mutated tumors, and not all DNA repair gene defects have the same impact across different cancer types. Alterations in *ATM*, *CDK12*, and *PALB2* were most commonly reported in prostate and breast cancer trials, whereas alterations in *RAD51C/D*, *CHEK2*, *RAD54L*, and *FANCM* were more frequently described in ovarian cancer studies (Supplementary Table S1). Exploratory gene-by-gene analyses were not always feasible, and comparisons may be confounded by multiple methodological and biological factors.

Regarding homologous recombination deficiency (HRD), recurrent genomic instability signatures following gene inactivation have been consistently observed for a limited subset of genes—namely, *PALB2*, *BRIP1*, *RAD51C*, *RAD51D*, and *BLM*—primarily in ovarian cancer. The evaluation of whether similar HRD-associated signatures can reliably predict PARP inhibitor sensitivity in other tumor types, such as breast, prostate, and pancreatic cancers, is currently under active investigation and remains to be fully established.

The present review identified several challenges that complicate the evaluation of the impact of HR gene defects on PARPi sensitivity. These include the rarity of events and limiting large meta-analysis. One of the limitations is the small number of these variant defects in each trial, especially as individual data related to the subgroup of patients harboring gene defect is scarce and not always clearly reported. A solution is to systematically and clearly report data related to these subgroups and all future trials related to PARPis. Another limitation found was that the extent of gene panels used in clinical trials were not standardized and were not all related to the HR core genes.

The same issue was found for variant interpretation; in fact, very few articles have reported clearly the criteria that were used in variant interpretation (multifactorial model, ACMG/AMP classification or experts variant database) [165,166,167,168,169,170,171]. Finally, the status of the *BRCA1/2* gene is sometimes partial: lack of the large rearrangement detection, mobile elements exploration and, above all, no epigenetic status [18,172,173,174,175,176,177]. The association with the HRD status should always be validated with the absence of any methylation either on the *BRCA1* or *RAD51C* promoting region [18] as they can fully explain the HRD status. Some association could be misled, for instance, in case of the methylation of *BRCA1*. Thus, future trials should clearly state this issue in the methodology knowing that variant interpretation could differ between databases. Expert gene level review is required, as HRD status alone should not currently be used as standalone evidence for variant classification [174]. This initiative is just beginning and is being launched exclusively for ovarian cancer [178].

### 5.1. Biological Determinants of Heterogeneous PARP Inhibitor Sensitivity

The heterogeneous clinical activity of PARP inhibitors (PARPis) among tumors harboring non-BRCA homologous recombination (HR) gene alterations likely reflects biological differences that extend beyond the presence of a pathogenic variant alone. Clinical trials reviewed in this study consistently demonstrated greater benefit in tumors with *PALB2*, *RAD51C*, and *RAD51D* pathogenic variants than in those harboring alterations in *ATM*, *CHEK2*, or *CDK12*, suggesting that not all HR genes confer equivalent PARPi sensitivity (Table 4). One important determinant is biallelic inactivation, as complete loss of gene function is generally required to establish a clinically relevant HR-deficient phenotype. Consequently, tumors carrying monoallelic pathogenic variants may retain sufficient HR activity to repair DNA damage, reducing susceptibility to PARPis. Because most clinical trials did not systematically assess loss of heterozygosity (LOH) or second-allele inactivation, this may partially explain the heterogeneous responses observed across studies [179].

The biological function of individual HR genes also influences treatment response. PALB2, RAD51C, and RAD51D are core components of the homologous recombination machinery and are directly involved in DNA repair, whereas ATM, CHEK2, and CDK12 primarily regulate DNA damage signaling, cell-cycle checkpoints, or transcription of DNA repair genes. Consequently, pathogenic variants in these latter genes may not consistently generate the profound HR deficiency required for synthetic lethality, which is consistent with the more modest clinical activity reported in trials such as PROfound, TOPARP-B, TALAPRO-1, GALAHAD, and TRITON2 [74,75,149,150].

The functional consequences of individual variants further contribute to response variability. Protein-truncating variants are generally more likely to impair HR than missense variants, although variant-specific functional effects are not routinely evaluated in clinical studies. Moreover, acquired resistance mechanisms—including secondary reversion mutations that restore HR function, replication fork protection, and activation of alternative DNA repair pathways—have been increasingly recognized as important causes of PARPi resistance following an initial response [180,181]. Collectively, these observations indicate that the predictive value of non-*BRCA HR* gene alterations depends not only on the affected gene but also on allele status, variant type, and functional consequences, reinforcing the need for integrated genomic and functional biomarkers, such as RAD51 foci assays or others, to improve patient selection.

### 5.2. Limitations of Current HRD Companion Diagnostic Assays

Although homologous recombination deficiency (HRD) assays have expanded the use of PARP inhibitors (PARPis) beyond *BRCA1/2*-mutated tumors, several limitations remain, particularly when evaluating pathogenic variants in non-*BRCA* homologous recombination (HR) genes. Most clinically validated companion diagnostic assays were originally developed using ovarian cancer cohorts enriched for *BRCA1/2* alterations, and their performance in tumors carrying defects in other HR genes is less well established [31,182].

The two assays most frequently used in clinical practice, MyChoice CDx and FoundationOne CDx, estimate HRD through different genomic approaches. MyChoice CDx integrates genomic instability parameters, including loss of heterozygosity (LOH), telomeric allelic imbalance, and large-scale state transitions into a genomic instability score (GIS), whereas FoundationOne CDx combines genome-wide LOH assessment with comprehensive genomic profiling. Because these platforms rely on different analytical methods and positivity thresholds, direct comparison of HRD status across clinical studies remains challenging [31,182,183].

Another important limitation is that genomic scar assays measure the genomic consequences of past HR deficiency rather than the current functional status of the repair pathway. Consequently, tumors that have restored homologous recombination through resistance mechanisms may remain HRD-positive, whereas tumors harboring pathogenic variants in non-*BRCA HR* genes may not accumulate sufficient genomic instability to be classified as HRD-positive. Moreover, most commercially available assays do not determine whether pathogenic variants are associated with **biallelic inactivation**, a key determinant of PARPi sensitivity. Future biomarker strategies will likely require integration of genomic scar assays with comprehensive genomic profiling and functional biomarkers, such as RAD51 foci formation, to improve prediction of PARPi benefit in tumors with non-*BRCA HR* gene alterations [53].

## 6. New Perspectives

The clinical benefit of PARPis in HRD tumors remains incompletely characterized. In many clinical trials, homologous recombination deficiency has been defined using composite genomic scores or loss-of-heterozygosity metrics, without systematically dissecting the individual contribution of PVs in specific HR genes. As a result, HRD is frequently treated as a binary phenotype, potentially obscuring biologically distinct mechanisms of genomic instability and heterogeneous responses to PARPis. Ongoing clinical studies aim to refine this framework by improving molecular stratification and better defining which HR alterations truly confer therapeutic vulnerability to PARPis (Tables S1–S6).

Among *non-BRCA* homologous recombination genes, *PALB2* has consistently emerged as the most promising candidate for clinical translation. Across multiple tumor types, including breast, ovarian, prostate, and pancreatic cancers, *PALB2* alterations have been associated with reproducible responses to PARPis, positioning this gene as the most likely next actionable target beyond *BRCA1/2*. This makes biological sense. These observations suggest that *PALB2* may represent an intermediate step toward expanding PARPi indications beyond classical HBOC-associated tumors [184].

In this context, combination strategies involving PARPis and immune checkpoint blockade are also being actively explored. A recent phase II study evaluated the combination of the PARPi niraparib with the anti-PD-1 antibody dostarlimab in patients with metastatic pancreatic cancer harboring germline or somatic *HRR* gene alterations, including *BRCA1/2*, *PALB2*, *RAD51C/D*, and *BARD1* (NCT04493060—Table S5). Although the study did not meet its primary endpoint of disease control rate at 12 weeks, exploratory analyses suggested differential activity according to mutation type, with higher disease control observed in patients with germline alterations and non-*BRCA2* pathogenic variants, particularly *BRCA1*. These results highlight both the biological complexity of HRD tumors and the current limitations of combining PARPis with immunotherapy in unselected or heavily pretreated populations, reinforcing the need for improved molecular stratification and predictive biomarkers [185].

Similar combination strategies are also being explored in breast cancer. A phase II pilot study evaluated the combination of olaparib with the anti-PD-1 antibody pembrolizumab in patients with advanced breast cancer harboring germline *BRCA* mutations or HRD (NCT03025035—Table S2). In this heavily pretreated population, the combination achieved an objective response rate of 33.3%, with manageable toxicity, including responses in patients previously exposed to PARP inhibitors. Although limited by small sample size and preliminary follow-up, these findings further support the biological rationale for combining PARP inhibition with immune checkpoint blockade in HRD tumors and underscore the need for larger, biomarker-driven studies to better define the patients most likely to benefit from this approach [186].

In line with this evolving strategy, additional phase II studies are being initiated to explore PARPi combinations in HRD-defined breast cancer populations, including an open-label trial evaluating fluzoparib in combination with the anti-PD-L1 antibody adebrelimab in HRD-positive HR+/HER2− advanced breast cancer (NCT06254066—Table S2).

Beyond PARPis, alternative therapeutic strategies targeting homologous recombination deficiency are also being explored. For instance, the phase Ib expansion study of CX-5461, a G-quadruplex-stabilizing agent, is currently investigating its activity in solid tumors harboring *BRCA1/2* or *PALB2* pathogenic variants or an HRD phenotype, while integrating exploratory biomarkers such as mutational signatures, circulating tumor DNA, and plasma DNA methylome profiling (NCT04890613—Table S6) [187].

Advances in sequencing technologies are also expected to significantly refine patient selection for PARP inhibitor therapy. Long-read sequencing approaches may allow the detection of complex genomic alterations, including structural variants and deep intronic pathogenic variants affecting homologous recombination genes, which are frequently missed by conventional short-read sequencing panels. Improved detection of such alterations may clarify currently unexplained HRD phenotypes and enhance the biological interpretation of HRD-positive tumors [176,188]. In addition, the integration of polygenic risk scores (PRSs) with tumor-based homologous recombination deficiency markers may further refine patient stratification for PARP inhibitor therapy, particularly in individuals without single high-penetrance pathogenic variants [189].

Notably, PARPis have also been tested for treatment of tumors beyond those classically related to HBOC syndrome harboring HR gene PVs, including lung, gastric, esophageal, urothelial, and endometrial cancers [2]. In the near future, as HRD assessment becomes more standardized and clinically validated, PARPi use may increasingly shift toward a biomarker-driven approach, in which treatment decisions are guided by HRD status rather than tumor type alone. However, this evolution is limited in fact as the threshold of HRD can be variable between tumors. In reviewing emerging data of PARPis and positive HRD tumors, several ongoing clinical trials were identified that are specifically evaluating this setting. Expansion of these cohorts will be essential to clarify the true clinical impact of PARPis in HDR tumors. Full transparency on the list of genes identified and the screening methodology will also be crucial.

Moreover, part of these ongoing trials evaluated new PARPis, such as Pamiparib, Fluzoparib, PARPis conjugated with microtubule polymerization inhibitor (e.g., drug AMXI-5001), and PARPis in association with a tankyrase inhibitor (e.g., drug JPI-547). Other combinations being evaluated are PARPis and immunotherapy, PARPis and different chemotherapy, and PARPis concomitant with radiotherapy treatment, such as the combination of Olaparib and radium-223 (Table S4). Notably, the majority of the trials consider response rate and progression-free survival as primary outcome, but also safety and tolerability, adverse events and dose limiting toxicity. Detailed characteristics of these ongoing studies, including tumor type, molecular selection criteria, and therapeutic strategies, are summarized in the Supplementary Material (Supplementary Tables S2–S6).

## 7. Conclusions

In conclusion, pathogenic variants in non-*BRCA* homologous recombination (HR) genes may serve as predictive biomarkers of response to PARP inhibitors (PARPis), although their clinical relevance varies according to the affected gene and tumor type. Among the non-*BRCA HR* genes, *PALB2* currently has the strongest clinical evidence supporting PARPi sensitivity, while *RAD51C* and *RAD51D* also demonstrate clinically meaningful benefit in selected settings. In contrast, the available evidence for *ATM*, *CHEK2*, *CDK12*, and other HR genes remains limited or inconsistent. Future prospective biomarker-driven studies integrating comprehensive genomic and functional HRD assessment are needed to refine patient selection and optimize the use of PARPis beyond *BRCA1/2*-associated cancers.

## Figures and Tables

**Table 1:** Frequency of somatic HR-gene alterations in prostate, ovarian, breast and pancreatic cancers [https://www.cbioportal.org/ (accessed on 25 March 2026)].

HR Genes	Prostate *n* = 6787	Ovary *n* = 1668	Breast *n* = 9062	Pancreas *n* = 1033
***ATM***	**4%**	1.4%	**2.5%**	**3%**
***ATR***	0.5%	0.2%	0.5%	0.6%
***ATRX***	0.4%	0.7%	0.4%	0.1%
***BARD1***	0.3%	0.1%	0.4%	0.1%
***BLM***	0.2%	0.4%	0.3%	0.4%
***BRIP1***	0.2%	0.2%	0.3%	0.3%
***CDK12***	**4%**	2.7%	0.8%	0.1%
***CHEK1***	0.5%	0.7%	0.6%	0%
***CHEK2***	1.5%	1%	0.3%	0.5%
***FANCA***	**2.7%**	**3%**	1.8%	0.3%
***FANCC***	0.2%	0.7%	0.2%	0.2%
***FANCD2***	1.2%	0.4%	0.8%	0.8%
***FANCE***	0%	0%	0%	0%
***FANCF***	0%	0%	0%	0%
***FANCG***	0%	0%	0%	0%
***FANCI***	0%	0%	0%	0%
***FANCL***	0.4%	0.1%	0.1%	0.2%
***FANCM***	0%	0%	0%	0%
***MRE11***	0.5%	0.6%	0.5%	0.1%
***NBN***	0.3%	0.5%	0.1%	0.4%
***PALB2***	0.4%	0.5%	0.2%	0.2%
***RAD50***	0.7%	0.5%	0.1%	0.7%
***RAD51***	0.6%	2.5%	0.5%	0.1%
***RAD51B***	0.4%	1.2%	0.6%	0.2%
***RAD51C***	0.5%	0.2%	0.4%	0.5%
***RAD51D***	0.4%	1.6%	0.2%	0.1%
***RAD52***	0%	0%	0%	0%
***RAD54L***	0%	0%	0%	0%
***RPA1***	0%	0%	0%	0%

**Table 2:** Selected preclinical studies of the association between *HR gene* defect and PARPi sensitivity of cancer cells.

Gene Symbol	Gene Alias	*In Vitro* Studies	*In Vivo* Studies	*HRD Status* [35]
*ATM*	Ataxia telangiectasia mutated serine/threonine kinase	[36,37,38,39,40,41,42,43]	[42,43]	HRP (*N* = 4)
*ATR*	Ataxia Telangiectasia and Rad3-Related Protein	[36,38,44,45]	[45,46]	ND
*ATRX*	Alpha thalassemia/intellectual disability syndrome X-linked	-	-	ND
*BARD1*	BRCA1-Associated RING Domain Protein 1	[47]	-	HRP (*N* = 1)
*BLM*	Bloom Syndrome RecQ Like Helicase	[39,48]	-	HRD (*N* = 2)
*BRIP1*	BRCA1 Interacting Protein C Terminal Helicase 1	[49]	-	HRD (*N* = 4)
*CDK12*	Cyclin-dependent kinase	[50,51]	-	HRP (*N* = 9)
*CHEK1*	Checkpoint Kinase 1	[36,38]	[46]	ND
*CHEK2*	Checkpoint Kinase 2	[36]	-	HRP (*N* = 3)
*FANCA*	Fanconi Anemia Complementation Group	[36]	-	HRP (*N* = 2)
*FANCC*	Fanconi Anemia Complementation Group	[36,39]	-	ND
*FANCD2*	Fanconi Anemia Complementation Group	[36,39,52]	[53]	ND
*FANCE*	Fanconi Anemia Complementation Group	-	-	ND
*FANCF*	Fanconi Anemia Complementation Group	-	-	ND
*FANCG*	Fanconi Anemia Complementation Group	[39]	-	ND
*FANCI*	Fanconi Anemia Complementation Group	-	-	HRP (*N* = 3)
*FANCL*	Fanconi Anemia Complementation Group	-	-	HRP (*N* = 2)
*FANCM*	Fanconi Anemia Complementation Group	-	-	HRP (*N* = 2)
*MRE11A*	Meiotic Recombination 11 Homolog 1	[54,55,56,57]	[57]	ND
*NBN*	Nijmegen Breakage Syndrome 1 (Nibrin)	[36,55]	-	HRP (*N* = 4)
*PALB2*	Partner And Localizer Of BRCA2	[58]	[53]	HRD (*N* = 3)
*RAD50*	RAD genes	[55]	-	ND
*RAD51(A)*	RAD genes	[36,44,59,60]	[60]	ND
*RAD51B*	RAD genes	-	-	HRP (*N* = 4)
*RAD51C*	RAD genes	[61]	[61]	HRD (*N* = 7)
*RAD51D*	RAD genes	[62]	-	HRD (*N* = 4)
*RAD52*	RAD genes	[48]	-	ND
*RAD54L*	RAD54 Like (DEAD-like helicase superfamily)	[36,48]	-	HRP (*N* = 1)
*RPA1*	Replication Protein A1	[36]	-	ND

**Table 3:** Clinical trials evaluating PARP inhibitor efficacy in tumors with homologous recombination gene alterations/pathogenic variants beyond *BRCA1/2*.

Clinical Trial (NCT/Name)	Phase	Cancer Type	PARP Inhibitor	BRCA Wild-Type Patients/HRD or Non-*BRCA HR* Gene Alterations	Reference
**NCT00753545 (Study 19)**	II	Recurrent platinum-sensitive OC	Olaparib	118/21	[139]
**NCT01847274 (ENGOT-OV16/NOVA)**	III	Recurrent platinum-sensitive OC	Niraparib	249/115 ‡	[140]
**NCT01891344 (ARIEL2)**	II	Relapsed platinum-sensitive HGOC	Rucaparib	154/20 *	[25]
**NCT01968213 (ARIEL3)**	III	Recurrent platinum-sensitive OC	Rucaparib	368/43 †	[141]
**NCT02477644 (PAOLA-1)**	III	Newly diagnosed advanced OC	Olaparib + bevacizumab	806/72 *	[142]
**NCT03402841 (OPINION)**	III	Platinum-sensitive relapsed OC	Olaparib	279/NR †	[143]
**NCT02983799 (LIGHT)**	II	Platinum-sensitive relapsed OC	Olaparib	270/NR †	[144]
**NCT03344965 (TBCRC-048)**	II	Metastatic breast cancer	Olaparib	36/36 *	[145]
**NCT02401347**	II	HER2-negative breast cancer or other solid tumors	Talazoparib	19/19 *	[146]
**NCT03140670**	II	Platinum-sensitive advanced pancreatic cancer	Rucaparib	2/2	[147]
**NCT02890355 (SWOG S1513)**	II	Metastatic pancreatic cancer	Veliparib	115/9	[148]
**NCT01682772 (TOPARP-B)**	II	mCRPC	Olaparib	66/66	[76]
**NCT02952534 (TRITON2)**	II	mCRPC	Rucaparib	78/78	[149]
**NCT02987543 (PROfound)**	III	mCRPC	Olaparib	227/227	[75]
**NCT03148795 (TALAPRO-1)**	II	mCRPC	Talazoparib	40/40 *	[150]
**NCT02854436 (GALAHAD)**	II	mCRPC	Niraparib	81/69	[151]
**NCT03732820 (PROpel)**	III	mCRPC	Olaparib	399/NR †	[152,153]

* Only a subset of enrolled patients were evaluable for response. † Non-*BRCA HR* gene alterations defined by loss of heterozygosity (LOH)-high status. NR † = not reported at gene level (non-*BRCA HR* genes) ‡ HRD-positive subgroup defined by genomic instability score according to study protocol. HGOC: high-grade ovarian cancer; OC: ovarian cancer; mCRPC: metastatic castration-resistant prostate cancer.

**Table 4:** Level of clinical evidence for PARP inhibitor sensitivity according to non-*BRCA* homologous recombination gene and tumor type.

Gene	Ovarian Cancer	Breast Cancer	Prostate Cancer	Pancreatic Cancer	Overall Evidence	Clinical Interpretation
*PALB2*	Moderate	Strong	Moderate	Moderate	Strong	Most consistent non-*BRCA* predictor of PARPi benefit across tumor types.
*RAD51C*	Moderate–Strong	Limited	Very limited	Not established	Moderate	Strongest evidence in ovarian cancer; limited data in other tumors.
*RAD51D*	Moderate–Strong	Limited	Very limited	Not established	Moderate	Similar to *RAD51C*, mainly supported in ovarian cancer.
*BRIP1*	Limited	Very limited	Very limited	Not established	Limited	Possible activity in selected cases, but insufficient for routine prediction.
*BARD1*	Very limited	Very limited	Very limited	Not established	Very limited	Evidence remains exploratory.
*FANCA*	Limited	Very limited	Limited	Very limited	Limited	Heterogeneous results; may require biallelic loss or functional HRD confirmation.
*NBN*	Limited	Very limited	Limited	Not established	Limited	Occasional responses reported, but evidence is sparse.
*RAD54L*	Limited	Not established	Very limited	Not established	Very limited	No consistent clinical signal.
*ATM*	Limited	Weak	Weak–Limited	Very limited	Weak/Inconsistent	Preclinical sensitivity does not consistently translate into clinical benefit.
*CHEK2*	Very limited	Weak	Weak	Not established	Weak/Inconsistent	Limited evidence for PARPi sensitivity; responses generally uncommon.
*CDK12*	Limited	Not established	Weak	Very limited	Weak/Inconsistent	Clinical activity appears modest and inconsistent, especially in prostate cancer.
*BLM*	Limited	Not established	Very limited	Very limited	Very limited	Mainly biological/preclinical rationale; clinical evidence insufficient.
*MRE11A*	Very limited	Not established	Very limited	Not established	Very limited	Preclinical evidence exists, but clinical validation is lacking.
*RAD50*	Very limited	Very limited	Very limited	Not established	Very limited	No consistent clinical evidence.
*FANCM*	Very limited	Not established	Very limited	Very limited	Very limited	Exploratory only.

**Strong:** reproducible clinical responses across studies and/or tumor types. **Moderate:** consistent signal in at least one tumor type, but limited by sample size or lack of prospective validation. **Limited:** occasional responses or subgroup signals with insufficient consistency. **Weak/Inconsistent:** low response rates or discordant results across trials. **Very limited/Not established:** evidence restricted to isolated cases, preclinical data, or no evaluable clinical data.

## Data Availability

Data sharing is not applicable to this article as no new data were created or analyzed in this study. All data supporting the findings of this study are derived from previously published studies and are appropriately cited within the article.

## Supplementary Materials

The following supporting information can be downloaded at: https://www.mdpi.com/article/10.3390/ijms27156754/s1.
